# Lipid metabolism-related genes are involved in the occurrence of asthma and regulate the immune microenvironment

**DOI:** 10.1186/s12864-023-09795-3

**Published:** 2024-02-01

**Authors:** Yuanmin Jia, Haixia Wang, Bin Ma, Zeyi Zhang, Jingjing Wang, Jin Wang, Ou Chen

**Affiliations:** 1 School of Nursing and Rehabilitation, Cheeloo College of Medicine, Shandong University, 44 Wenhua Xi Road, Lixia District, Jinan City, Shandong Province China; 2 Department of Pediatrics, Jinan Maternity and Child Care Hospital, No. 2, Jianguo Xiaojing 3Rd Road, Shizhong District, Jinan City, Shandong Province China

**Keywords:** Asthma, Lipid metabolism, Immune microenvironment, WGCNA, Competing endogenous RNA, Diagnostic biomarker

## Abstract

**Background:**

Lipid metabolism plays a pivotal role in asthma pathogenesis. However, a comprehensive analysis of the importance of lipid metabolism-related genes (LMRGs) in regulating the immune microenvironment in asthma remains lacking. The transcriptome matrix was downloaded from the Gene Expression Omnibus (GEO) dataset. Differentially expressed analysis and weighted gene coexpression network analysis (WGCNA) were conducted on the GSE74986 dataset to select hub LMRGs, and gene set enrichment analysis (GSEA) was conducted to explore their biological functions. The CIBERSORT algorithm was used to determine immune infiltration in the asthma and control groups, and the correlation of diagnostic biomarkers and immune cells was performed via Spearman correlation analysis. Subsequently, a competitive endogenous RNA (ceRNA) network was constructed to investigate the hidden molecular mechanism of asthma. The expression levels of the hub genes were further validated in the GSE143192 dataset, and RT‒qPCR and immunofluorescence were performed to verify the reliability of the results in the OVA asthma model. Lastly, the ceRNA network was confirmed by qRT-PCR and RNAi experiments in the characteristic cytokine (IL-13)-induced asthma cellular model.

**Results:**

*ASAH1*, *ACER3* and *SGPP1* were identified as hub LMRGs and were mainly involved in protein secretion, mTORC1 signaling, and fatty acid metabolism. We found more infiltration of CD8^+^ T cells, activated NK cells, and monocytes and less M0 macrophage infiltration in the asthma group than in the healthy control group. In addition, *ASAH1*, *ACER3*, and *SGPP1* were negatively correlated with CD8^+^ T cells and activated NK cells, but positively correlated with M0 macrophages. Within the ceRNA network, *SNHG9*-*hsa-miR-615-3p*-*ACER3*, *hsa-miR-212-5p* and *hsa-miR-5682* may play crucial roles in asthma pathogenesis. The low expression of *ASAH1* and *SGPP1* in asthma was also validated in the GSE74075 dataset. After *SNHG9* knockdown, *miR-615-3p* expression was significantly upregulated, while that of *ACER3* was significantly downregulated.

**Conclusion:**

*ASAH1*, *ACER3* and *SGPP1* might be diagnostic biomarkers for asthma, and are associated with increased immune system activation. In addition, *SNHG9*-*hsa-miR-615-3p*-*ACER3* may be viewed as effective therapeutic targets for asthma. Our findings might provide a novel perspective for future research on asthma.

**Supplementary Information:**

The online version contains supplementary material available at 10.1186/s12864-023-09795-3.

## Introduction

Bronchial asthma (asthma) is a chronic inflammatory disease caused by complex gene‒environment interactions, and is characterized by chronic airway inflammation, airway hyperresponsiveness, mucus hypersecretion and airflow obstruction [1]. Approximately 300 million individuals worldwide are affected by asthma, with prevalence rates ranging from 1 to 18% in different countries [1, 2]. It is an intractable heterogeneous and multifactorial disease with a wide range of molecular, biochemical and cellular inflammatory characteristics. Although effective therapies that could relieve asthma symptoms are available, a large proportion of patients show poor control and experience persistent residual symptoms, indicating that the underlying pathogenetic mechanisms of asthma are unclear [3, 4]. Recently, lipid metabolism has been regarded as a novel hallmark of asthma [5]. Therefore, identifying the potential biomarkers of lipid metabolism and mechanisms underlying asthma might provide insights into the pathogenesis of asthma and determine new therapeutic targets.

Lipid metabolism is a complex physiological process including the uptake, transport, biosynthesis (anabolism) and degradation (catabolism) of lipids [6], that participates in many active functions of our body, such as energy storage, nerve impulse transmission, hormone regulation, protein distribution and function, and cell inflammation [6, 7]. Increasing evidence from laboratory and clinical studies has indicated that lipid metabolism plays a pivotal role in the pathogenesis of asthma [5]. For example, fatty acid metabolism could activate macrophages, leading to the generation of various inflammatory cytokines, such as TNF-α, IL-6, and IL-1β, which impact lung function and participate in the initiation of asthma in patients with obesity [8]. McErlean et al [9]. proved that prostaglandin-endoperoxide synthase 1 (PTGS1), a lipid metabolism-associated enzyme, was implicated in epigenetic mechanisms underlying asthma pathogenesis in the airways. A laboratory study showed that lipid metabolism-related genes, such as *Scd1*, *Fasn*, and *Lpcat1*, downstream of the STAT3-SCD1 axis contributing to lung homeostasis could suppress allergic airway inflammation in asthma models [10]. In addition, lipid metabolism regulates many immune cellular processes, such as IgE production by B cells, eosinophil migration to the lungs, and perturbations in the Th1/Th2 balance, which in turn affect allergic asthma [5]. The specific knockout of PGI_2_ analogs or PGI_2_ receptors upregulates IL-4, IL-5, and IL-13 release from T cells in vitro and in vivo and ultimately aggravates asthma [11, 12]. Although, existing studies have revealed the significant roles of lipid metabolism and lipid metabolism-related genes (LMRGs) in asthma, most studies have focused on specific LMRGs, and studies comprehensively analysing LMRGs in combination with the immune microenvironment of asthma have rarely been conducted.

Based on this, we conducted differential expression analysis and weighted gene coexpression network analysis (WGCNA) and further constructed a competitive endogenous RNA (ceRNA) network to select hub LMRGs and reveal the hidden intrinsic molecular mechanism of hub LMRGs in asthma. Additionally, immune infiltration analysis and correlation analysis between hub LMRGs and immune cells were performed to explore the relationships between hub LMRGs, the immune microenvironment, and asthma. Our study might provide novel diagnostic biomarkers and therapeutic targets for asthma and promote the personalized treatment of asthmatic patients.

## Materials and methods

### Data acquisition and processing

We downloaded asthma microarray datasets from the Gene Expression Omnibus (GEO) database (https://www.ncbi.nlm.nih.gov/geo/). The selection criteria included the following: i) the gene expression profiling must include both disease and control groups; ii) asthma was defined by the criteria of the Global Initiative for Asthma (GINA) [2] and the national asthma guidelines; and iii) samples had a mapped gene expression matrix. Profiles with nonhuman tested specimens, incomplete data, related to cell lines, and associated with other diseases were excluded. Four datasets with reliable sample sources were included in this study, including 2 mRNA (GSE74986 and GSE74075) [13, 14], 1 miRNA (GSE120172) [15], and 1 lncRNA (GSE143192) [16] expression profiles. The GSE74986 dataset annotated using GPL6480 (Agilent-014850 Whole Human Genome Microarray 4 × 44 K G4112F) contained bronchial-alveolar lavage samples from 74 asthmatic patients and 12 healthy controls [13]; the GSE74075 dataset used as the validation set contained 16 samples (asthma:normal = 10:6); the GSE120172 dataset involved 24 samples (asthma:normal = 12:12); and the GSE143192 dataset involved 8 samples (asthma:normal = 4:4). More detailed information about the four datasets and the characteristics of the participants are shown in Additional file 1: Table S1 and Table S2.

The raw microarray gene expression data were preprocessed by the R Bioconductor package affy, including background correction, normalization, and log2 transformation [17]. The microarray probes were converted into gene symbols based on the platform annotation file, probe sets without corresponding gene symbols were removed, and average expression values were retained when one gene was targeted by several probes. In addition, a total of 769 LMRGs were obtained from the molecular signatures database (MSigDB) (https://www.gsea-msigdb.org/gsea/msigdb).

### Differentially expressed genes identification

Differentially expressed genes (DEGs) between asthmatic patients and healthy controls from the GSE74986 dataset were selected utilizing the “limma” package in R [18]. The Benjamini–Hochberg (FDR) [19] procedure was used to correct for multiple testing. FDR < 0.05 and |log2(fold change, FC)|> 1 were set as cut-off thresholds for statistical significance.

### Identification of gene modules by weighted gene coexpression network analysis (WGCNA)

WGCNA has been extensively used in investigating the relationship between coexpression gene modules and clinical phenotypes [20]. We utilized the R package “WGCNA” to construct coexpression networks [20]. 1) Genes with greater than 25% variation were selected for WGCNA. 2) The hclust function was utilized to cluster samples and recognize outliers. 3) The soft-thresholding power, which was derived by coexpression similarity, was calculated using the pick-Soft-Threshold function of WGCNA. The optimal power was chosen when the scale-free index (R^2^) reached 0.80 and the mean connectivity approximated 0. 4) The topological overlap matrix (TOM) and the corresponding dissimilarity (1-TOM) were transformed from the adjacency matrix. 5) Modules were detected through hierarchical clustering and dynamic tree cut function, with a minimum number (gene group) of 50 for the genes dendrogram [21]. 6) For the modules correlated with the clinical phenotypes, gene significance (GS) and module membership (MM) were calculated and visualized. Finally, the module with the highest correlation coefficient was selected as the key module and applied for further analysis.

### Identification of BA-lipid metabolism-related DEGs (BA-LM DEGs) and functional enrichment analysis

The BA-LM DEGs were obtained from the intersection of the LMRGs, WGCNA significant module genes, and DEGs detected from the GSE74986 dataset using the “VennDiagram” package in R. The differentially expressed BA-LM DEGs between asthmatic patients and healthy controls were identified by the Wilcoxon test. To explore the potential roles of the BA-LM DEGs in asthma, ClueGO, a Cytoscape plug-in, was used to perform Gene Ontology (GO) and Kyoto Encyclopedia of Genes and Genomes (KEGG) enrichment analyses, and create a functionally organized GO/pathway term network [22]. A *p* value < 0.05 was regarded as the cut-off threshold.

### Identification of hub genes and gene set enrichment analysis (GSEA)

The STRING website (https://cn.string-db.org/) was used to build a protein–protein interaction (PPI) network of BA-LM DEGs detecting gene connections, and minimum confidence ≥ 0.4 was defined as the cut-off threshold [23]. The network was then visualized with Cytoscape software (version 3.9.1) [24], after removing discrete proteins. We used the molecular complex detection (MCODE) plug-in in Cytoscape software to extract the pivotal subnetwork and obtain hub genes [25]. Additionally, gene set enrichment analysis (GSEA) with the annotation of hallmark gene sets was conducted via the SangerBox platform (http://sangerbox.com/) to analyse the biological functions of hub genes in asthma [26]. Asthmatic patients were divided into low- or high-expression groups based on the median value of hub gene expression, and GSEA was performed with nominal *P* < 0.05, |enrichment scores| (ES) > 0.4, and false discovery rate (FDR) < 0.25 considered statistically significant [27].

### Immune cell infiltration analysis

To uncover the immune infiltration landscape of asthma and control samples, the “CIBERSORT” R package, which could calculate the abundance of specific cells in the mixture matrix, was used [28]. The proportions and heatmap of the 22 immune cells in the samples were visualized using the “barplot” and “pheatmap” packages, respectively. Subsequently, the “vioplot” package was used to compare the differences in infiltrating immune cells between the two groups [29], and the “Corrplot” package was further used to draw a correlation heatmap of 22 immune cells. Finally, Spearman correlation analysis of hub genes and immune cells was performed via the SangerBox platform (http://sangerbox.com/), and a *P* value < 0.05 was set as the cut-off criterion.

### Construction of the ceRNA network

Differentially expressed miRNAs (DEmiRNAs) and lncRNAs (DElnRNAs) between asthma and control samples from the GSE120172 and GSE143192 datasets, respectively, were analysed by the “limma” R package [18], upon selection criteria of *p* value < 0.05 and |log2(fold change, FC)|> 0.5. Then, we utilized miRWalk (http://mirwalk.umm.uni-heidelberg.de/) combining the TargetScan, miRDB and miRTarBase databases to determine the target miRNAs of hub genes [30]. Target miRNAs were intersected with DEmiRNAs from GSE120172 to obtain common miRNAs. Next, StarBase (http://starbase.sysu.edu.cn/starbase2/index.php) was used to predict lncRNAs interacting with the common miRNAs [31], and the common lncRNAs were identified by taking the intersection of the target lncRNAs and DElncRNAs from GSE143192. Finally, we integrated hub genes, miRNAs, and lncRNAs to construct a lncRNA‒miRNA-mRNA ceRNA network and visualized it using Cytoscape software [24] (version 3.9.1).

### Validation in the GEO dataset

The present study confirmed the expression status of potential hub genes from the GSE74075 dataset using the Wilcoxon test method with a *p* value < 0.05 as the critical value to consider statistical significance.

### Validation in the OVA models

#### Animals and grouping

C57BL/6 J male mice were provided by Vital River Laboratories (Beijing, China). Mice were allowed tap water and rodent chow and were maintained on a 12 h light/dark cycle under a favorable environment (22–24℃). After 1 week of acclimatization, the C57BL/6 mice were randomly divided into two groups (*n* = 4 per group). The OVA group was sensitized by an i.p. injection (100 µL) of 20 µg chicken OVA (Sigma, United States) emulsified in Imject alum (Pierce, United States) on days 0 and 14 and subsequently challenged for 40 min with an aerosol generated by ultrasonic nebulization of 2% OVA in saline from 24 to 41 days [32]. The control group was treated with saline in both the sensitization and excitation phases. All experimental procedures used in this study were approved and conducted according to the guidelines of the laboratory Animal Management Committee of Shandong University.

### Real-time quantitative polymerase chain reaction (RT‒qPCR)

Total RNA was extracted from lung tissues using TRIzol reagent (Cwbio, Jiangsu, China) following the manufacturer’s instructions. Then, the extracted RNA was reverse-transcribed into cDNA using the HiFiScript cDNA Synthesis Kit (Cwbio). RT‒qPCR was performed using an UltraSYBR Mixture (Cwbio) on real-time PCR detection equipment (Bio-Rad, Hercules, CA, United States). The primer sequences were as follows: mouse *ASAH1* (Forward: 5’-AGTCTTCTCACCTGGGTCCTA-3’, Reverse: 5’-CAATCTTCTGTCCACGGCGG-3’), mouse *ACER3* (Forward: 5’-TGCATGTTTGAGTGTTTCAAGA-3’, Reverse: 5’-ACCAACATTCCATACATGACCTG-3’), mouse *SGPP1* (Forward: 5’-GCTTGTACTGTTCGTGAGGGA-3’, Reverse: 5’-GAACCCAACCATCCCGTAGG-3’), and mouse GAPDH (Forward: 5’-GGCCCCTCTGGAAAGCTGTGG-3’, Reverse: 5’-CCCGGCATCGAA GGT-GGAAGA-3’) were purchased from Sangon Biotech (Shanghai, China). GAPDH served as an internal reference for mRNAs and lncRNAs, and U6 was employed as an internal reference for miRNAs. The expression differences between groups were compared using the *t* test (two-tailed) in GraphPad Prism 9. *P* < 0.05 was considered statistically significant. Detailed sequences are listed in Additional file 2: Table S3.

### Immunofluorescence staining

For immunofluorescent (IF) staining, lung tissues were fixed with 4% paraformaldehyde for 20 min. Samples were incubated with anti-rabbit ASAH1 antibody (1:80, Proteintech, Chicago, United States), anti-rabbit ACER3 (1:200, Proteintech, Chicago, United States), and anti-rabbit -SGPP1 antibody (1:200, Affinity, Ancaster, Canada). The secondary antibody was coralite 594-conjugated goat anti-rabbit IgG (H + L) (1:200, Proteintech, Chicago, United States). Nuclei were dyed with 4,6 diamidino-2-phenylindole (DAPI) (Sigma, Darmstadt, Germany). All the above staining was conducted according to the manufacturer’s instructions. Images were observed and captured with a fluorescence microscope (Nikon, Tokyo, Japan). Image analysis was performed with ImageJ (NIH, Bethesda, MD, USA) and GraphPad Prism 9 software (San Diego, CA, USA).

### Cell culture and transfection

BEAS-2B cells were purchased from the Cell Bank of the Chinese Academy of Sciences and were cultured in DMEM high glucose medium (CM15019, Macgene) containing 0.1% antibiotics (BL505A, Biosharp) and 10% FBS (A6907FBS-500, Invigentech) at 37 °C in a humidified atmosphere of 5% CO_2_. BEAS-2B cells were seeded in 6-well plates and grown to 60–70% confuence, at which time media were exchanged for antibiotic-free media. Cells were then transfected with si-*SNHG9* (General Biol, Chuzhou, China) (sequence: 5'-CCCGAAGAGUGGCUAUAAATT-3') using the transfection reagent (OGTR(C)20131001, Obio, Shanghai, China), in accordance with the manufacturer’s protocol. At 6 h post-transfection, BEAS-2B cells were then treated with Human IL-13 (C-Fc) (C01M, Novoprotein, Suzhou, China) for 24 h to establish an asthma cell model.

## Results

### Identification of DEGs

**Fig. 1:**
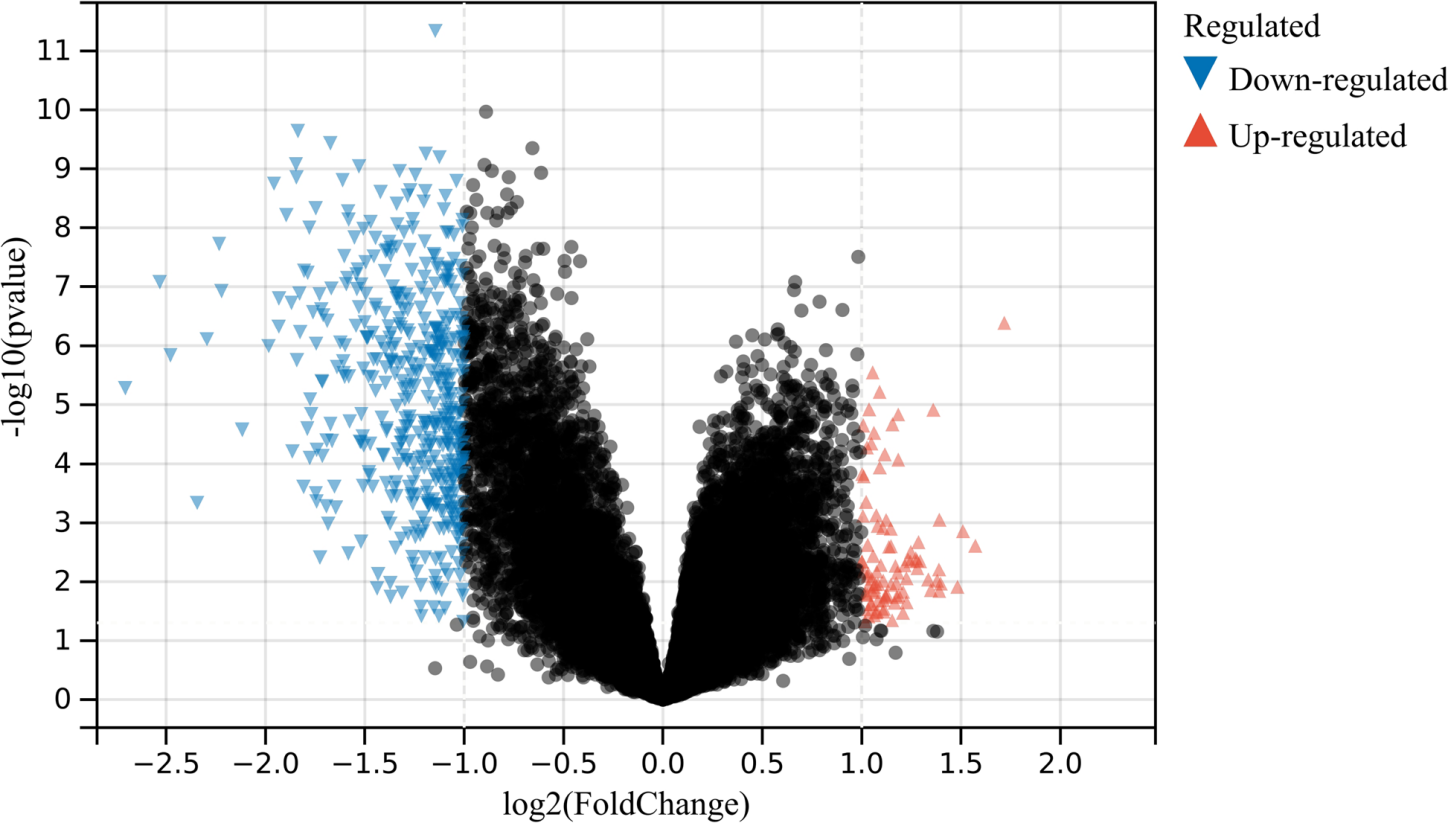
Volcano diagram showing DEGs between asthma and control groups. DEGs: differentially expressed genes

**Fig. 2:**
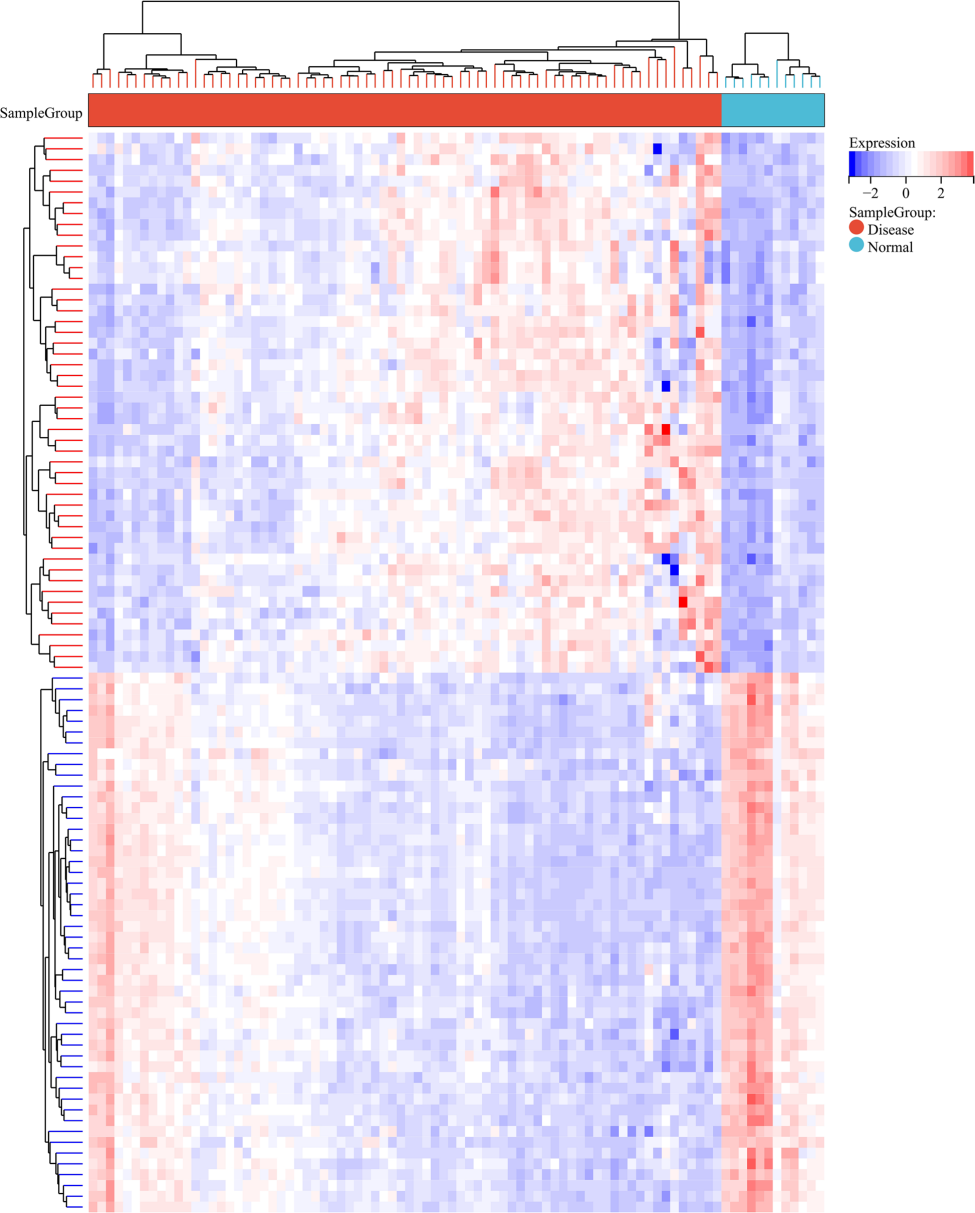
Heatmap of top 50 up-regulated and down-regulated DEGs. DEGs: differentially expressed genes

A total of 520 DEGs between asthma and control samples were detected in the GSE74986 dataset under the cut-off thresholds of FDR < 0.05 and |log2(fold change, FC)|> 1, among which 64 genes were upregulated and 456 genes were downregulated (Additional file 3: Table S4 and Additional file 4: Table S5). The volcano plot of 520 DEGs and heatmap of the top 50 DEGs are shown in Figs. 1 and 2 respectively.

### Identification of gene modules by WGCNA

**Fig. 3:**
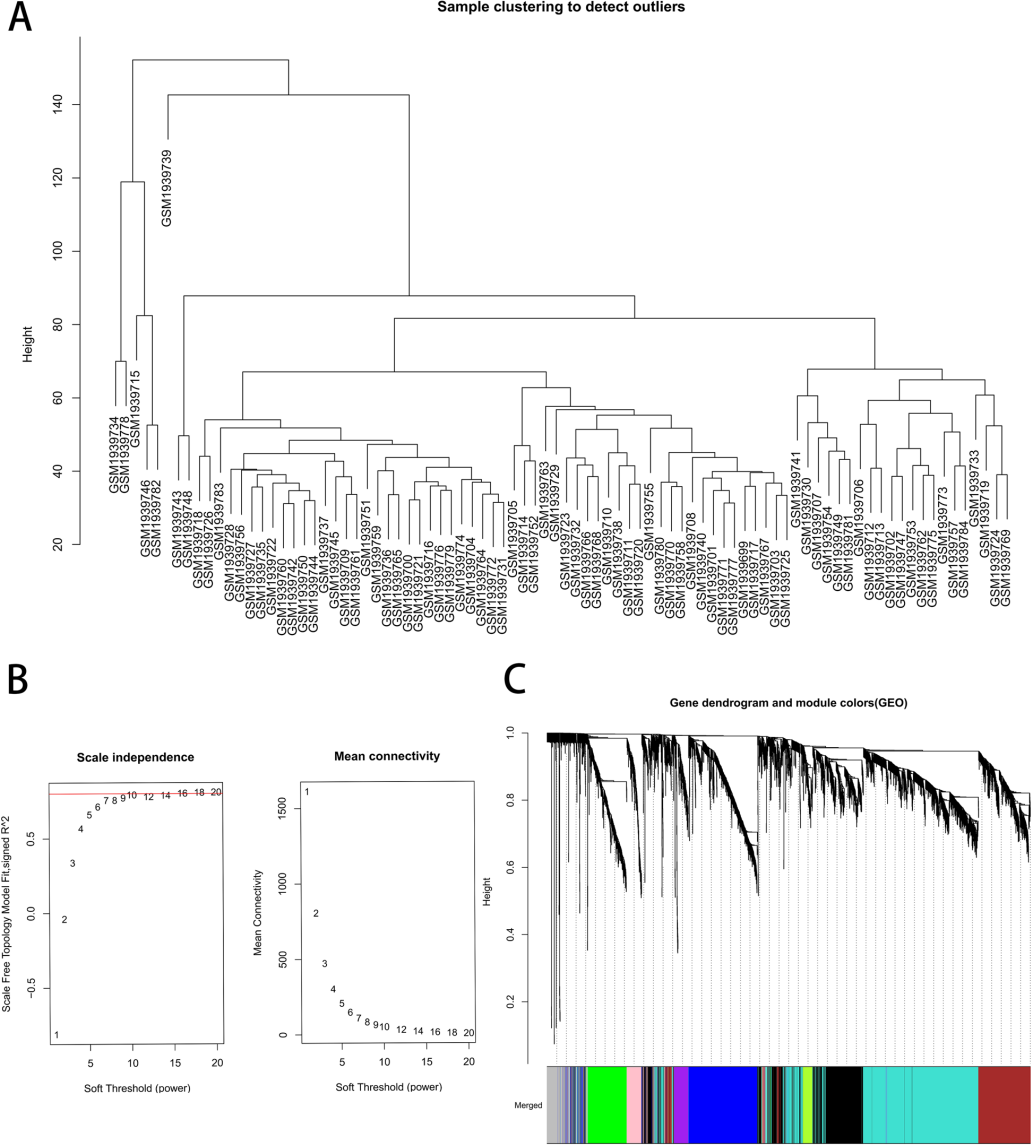
Weighted gene co-expression network analysis (WGCNA). **A** Clustering dendrogram of samples. **B** Analysis of network topology for various soft-thresholding powers. **C** Clustering dendrogram of genes

**Fig. 4:**
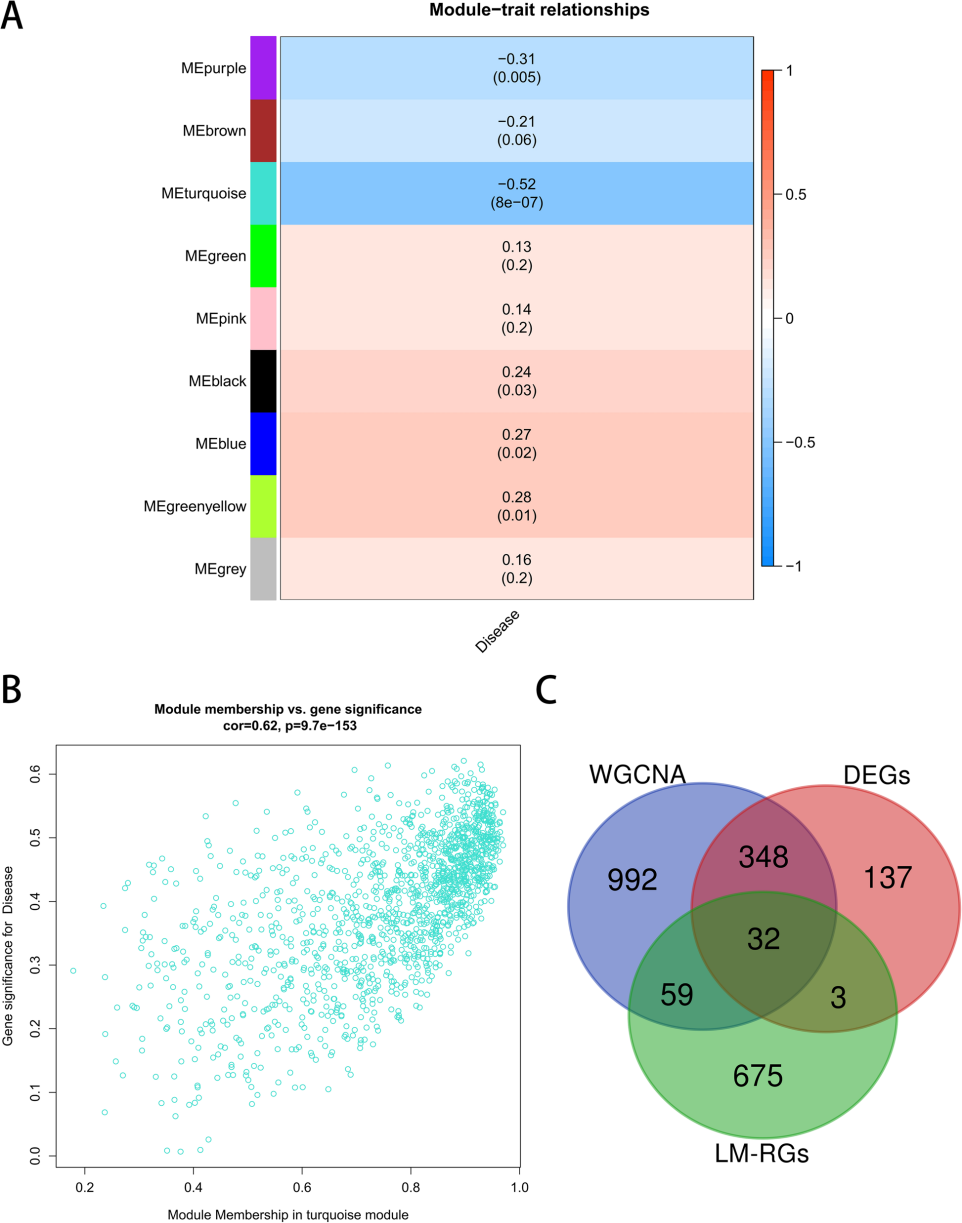
WGCNA and Venn diagram. **A** Heatmap of the association between modules and asthma. **B** Correlation plot between MM (X-axis) and GS (Y-axis) of genes contained in the turquoise module. **C** Venn diagram showing common genes between the DEGs, LMRGs and the genes in the turquoise module. MM: module membership, GS: gene significance, DEGs: differentially expressed genes, LMRGs: lipid metabolism related genes, WGCNA: Weighted gene co-expression network analysis

Cluster analysis was performed to detect and remove outliers, the height cut-off value was set at 160, and all samples were retained for subsequent analysis (Fig. 3A). The optimal soft threshold power was determined to be 10 (scale-free *R*^2^ = 0.80) to ensure a scale-free network (Fig. 3B). The dynamic shear tree algorithm was used to merge modules (dissimilarity degree < 25%) at the minimum module size of 50, and a total of 9 modules were obtained, in which genes had similar coexpression traits (Fig. 3C). The correlation analysis between modules and traits demonstrated that the turquoise module exhibited the highest adverse correlation with asthma (cor = -0.52; *P* < 0.001) (Fig. 4A and Additional file 5: Table S6), and Fig. 4B shows that a strong GS-MM correlation was obtained in the turquoise module (cor = 0.62, *p* < 0.001). Therefore, the turquoise module was considered the key module, in which the downregulated genes were regarded as BA-related genes.

### Identification of BA-LM DEGs and functional enrichment analysis

**Fig. 5:**
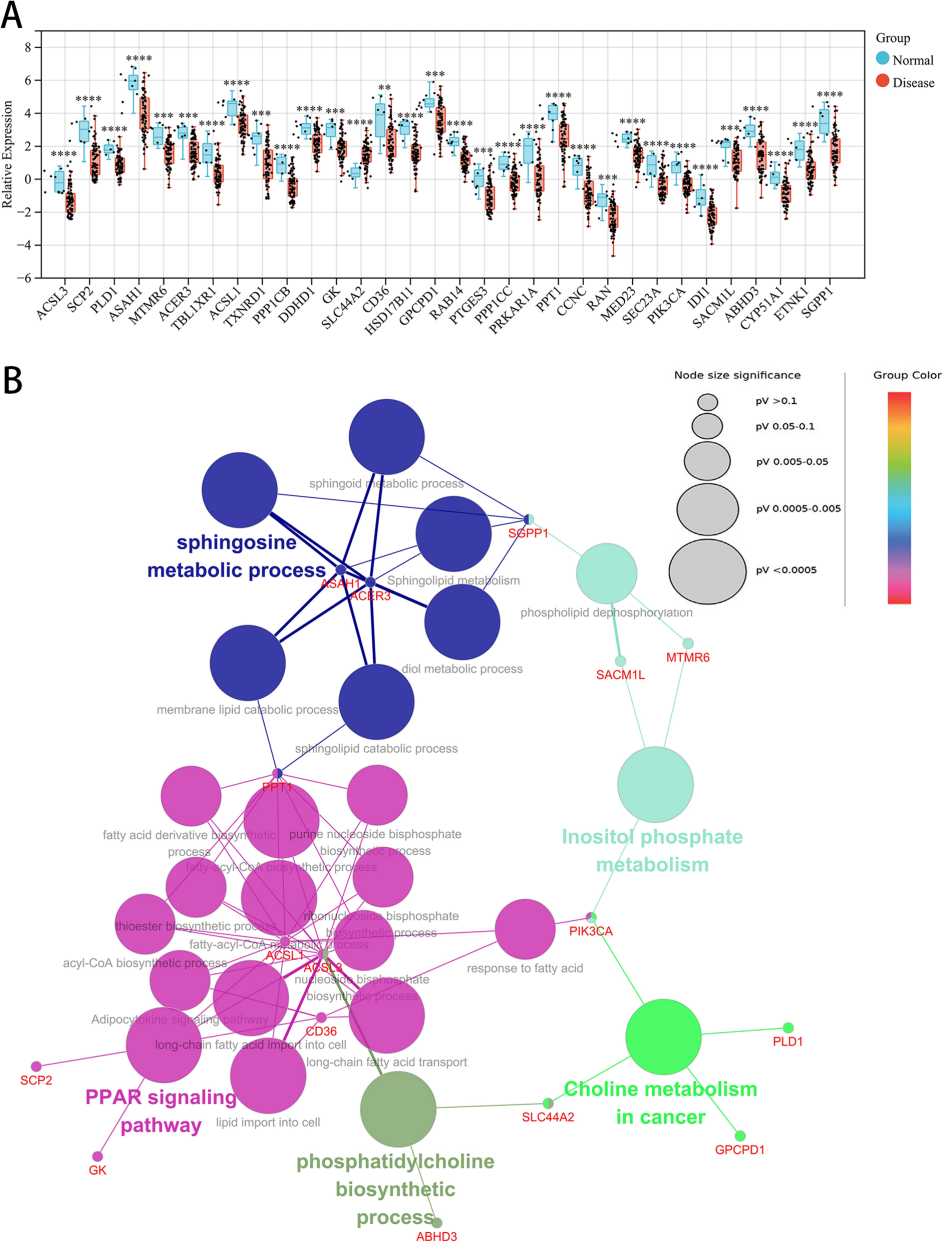
Identifcation and enrichment analysis of common genes. **A** Boxplot of 32 common genes in asthma and control groups. **B** Functional enrichment of 32 common genes. **p < .01, ***p < .001, and ****p < .0001

As shown in the Venn diagram (Fig. 4C), 32 BA-LM DEGs were identified by intersecting 769 LMRGs, 1431 WGCNA significant module genes, and 520 DEGs. The comparison analysis revealed that asthmatic patients had lower expression of *ABHD3*, *ACER3*, *ACSL1*, *ACSL3*, *ASAH1*, *CCNC*, *CD36*, *CYP51A1*, *DDHD1*, *ETNK1*, *GK*, *GPCPD1*, *HSD17B11*, *IDI1*, *MED23*, *MTMR6*, *PIK3CA*, *PLD1*, *PPP1CB*, *PPP1CC*, *PPT1*, *PRKAR1A*, *PTGES3*, *RAB14*, *RAN*, *SACM1L*, *SCP2*, *SEC23A*, *SGPP1*, *TBL1XR1*, and *TXNRD1* and higher expression of *SLC44A2* (Fig. 5A). GO and KEGG enrichment analyses showed that BA-LM DEGs were mainly enriched in the PPAR signaling pathway, sphingosine metabolic process, and inositol phosphate metabolism. Further investigation revealed that *ACER3* and *ASAH1* were mainly involved in sphingolipid metabolism, diol metabolic process, membrane lipid catabolic process, sphingolipid catabolic process, sphingoid metabolic process, and sphingosine metabolic process. *SGPP1* was closely related to phospholipid dephosphorylation, sphingolipid metabolism, diol metabolic process, sphingoid metabolic process, and sphingosine metabolic process (Fig. 5B).

### Identification of hub genes and GSEA

**Fig. 6:**
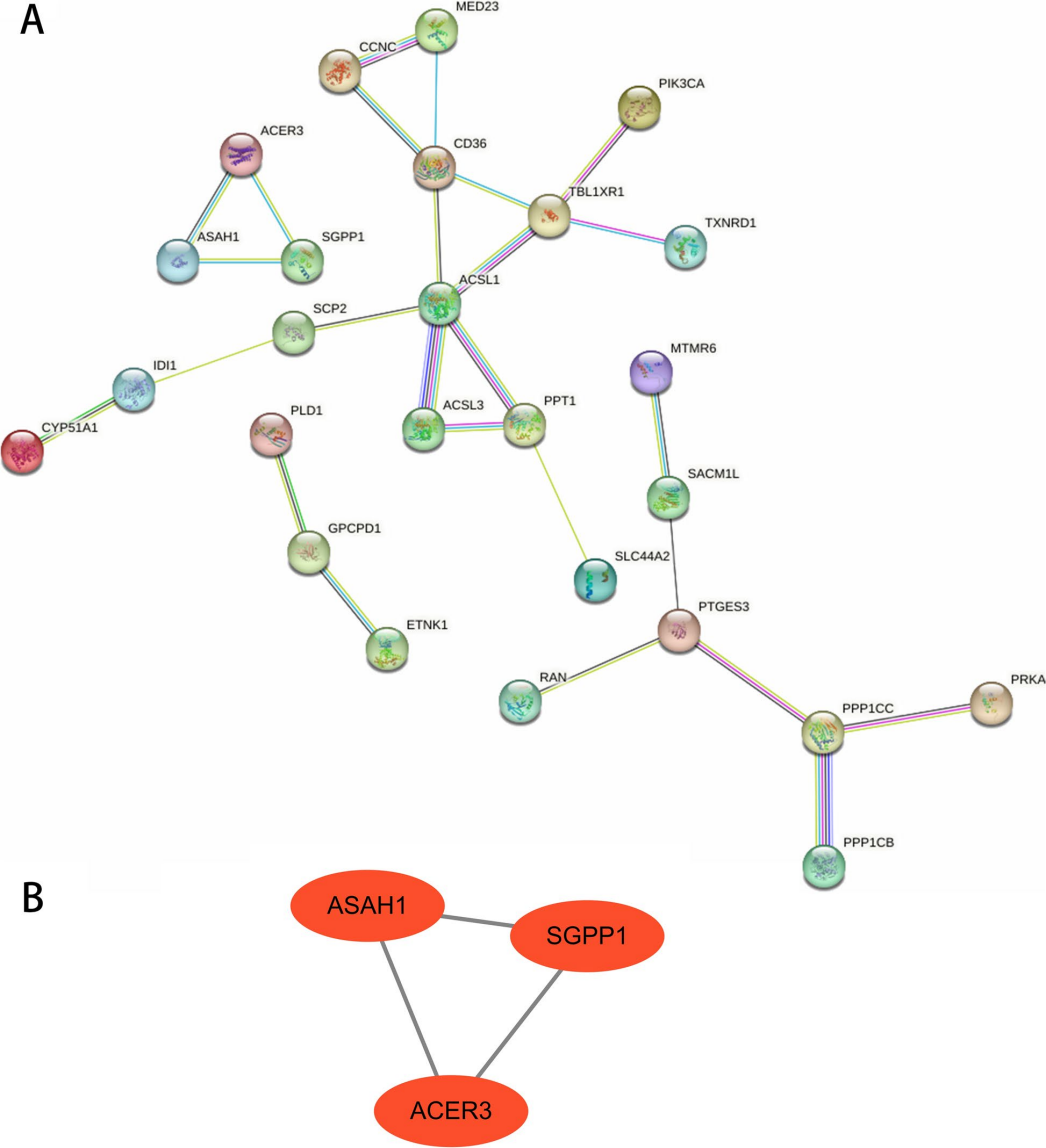
PPI network and the hub genes. A Protein–protein interaction network. B Hub genes identifed from the PPI network using the MCODE. PPI: protein–protein interaction, MCODE: molecular complex detection

**Fig. 7:**
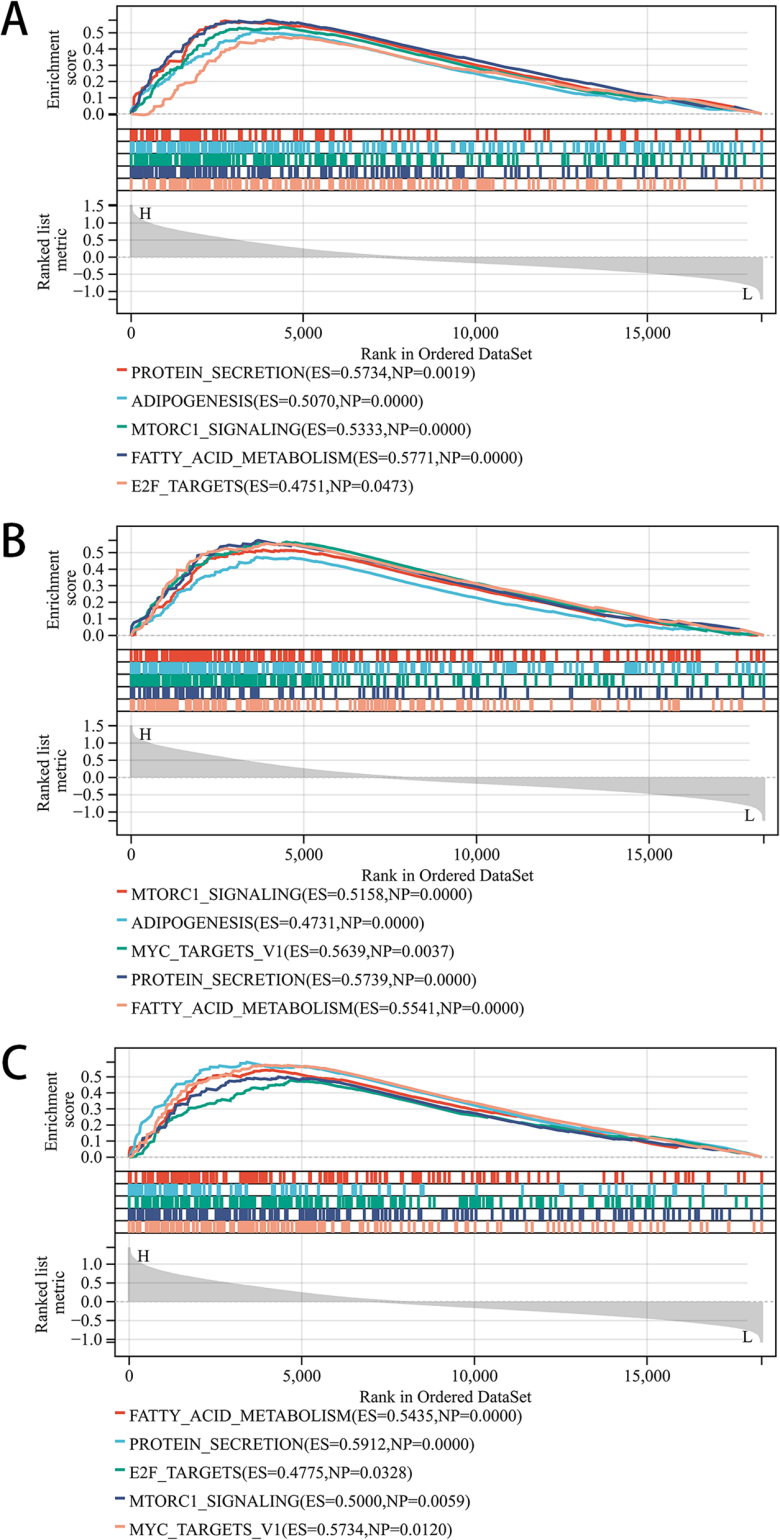
GSEA enrichment analysis. **A**-**C** GSEA enrichment plots of hub genes (*ASAH1*, *ACER3* and *SGPP1*). GSEA: gene set enrichment analysis

After removing discrete proteins, a PPI network was created with 26 nodes and 26 edges that contained 25 downregulated genes and 1 upregulated gene (Fig. 6A). The MCODE analysis identified a key module in the network, including three hub genes (*ASAH1*, *ACER3* and *SGPP1*), all of which were downregulated genes and strongly linked to asthma (Fig. 6B). To deeply analyse the effects of hub genes on asthma, we performed GSEA to analyse the potential signaling pathways. The top five hallmark pathways are shown in Fig. 7A-C. Protein secretion, adipogenesis, mTORC1 signaling, fatty acid metabolism, and E2F targets were significantly enriched in *ASAH1* high-expression samples. Moreover, mTORC1 signaling, adipogenesis, MYC target v1, protein secretion, and fatty acid metabolism were significantly influenced by increased *ACER3* expression in asthma. *SGPP1* high-expression samples were predominantly enriched in fatty acid metabolism, protein secretion, E2F targets, mTORC1 signaling, and MYC target v1.

### Immune cell infiltration analysis

**Fig. 8:**
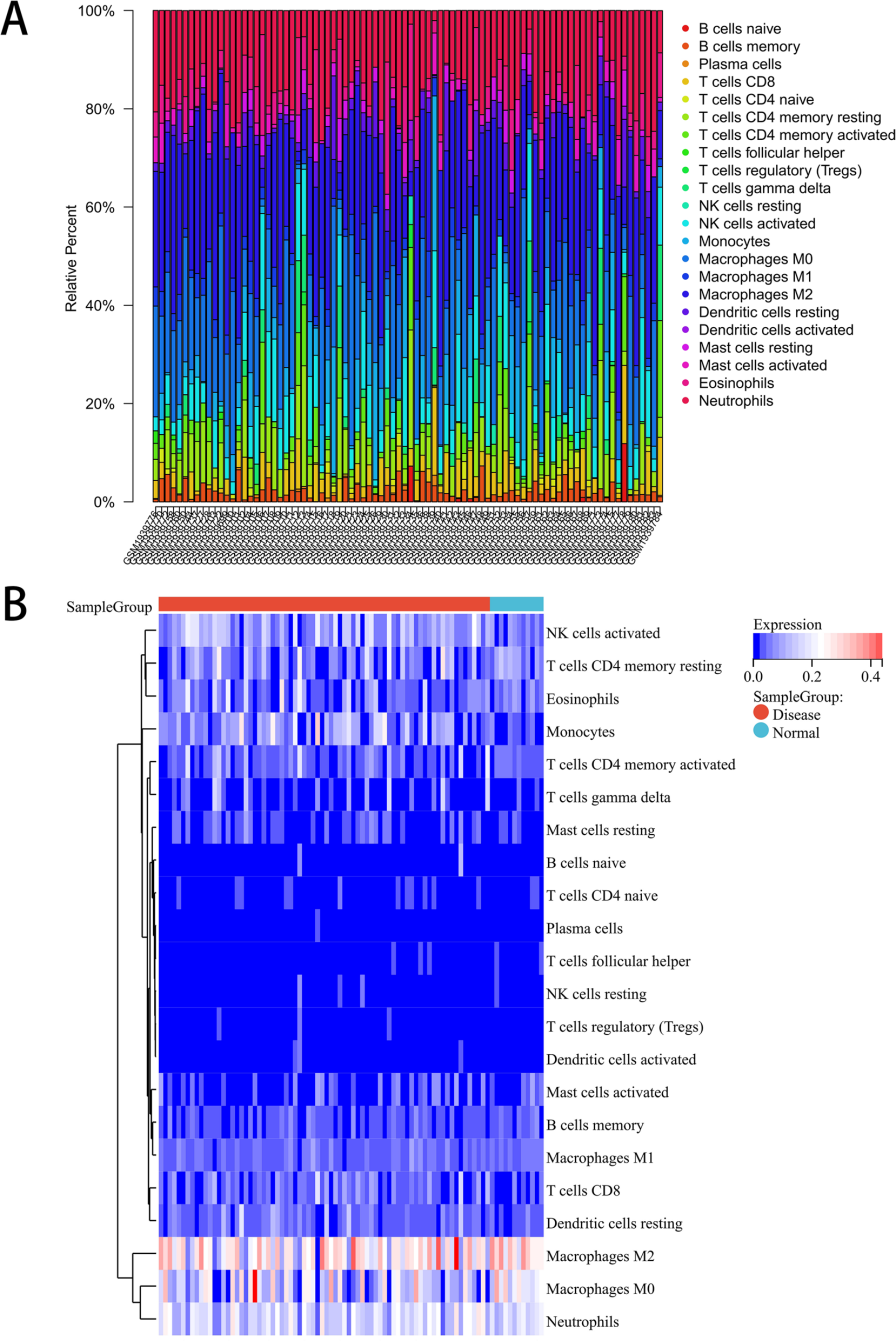
The landscape of immune infiltration between asthma and control groups. **A** Pile-up histogram showing composition of immune cells in each sample. **B** Heatmap displaying the distribution of 22 types of immune cells

**Fig. 9:**
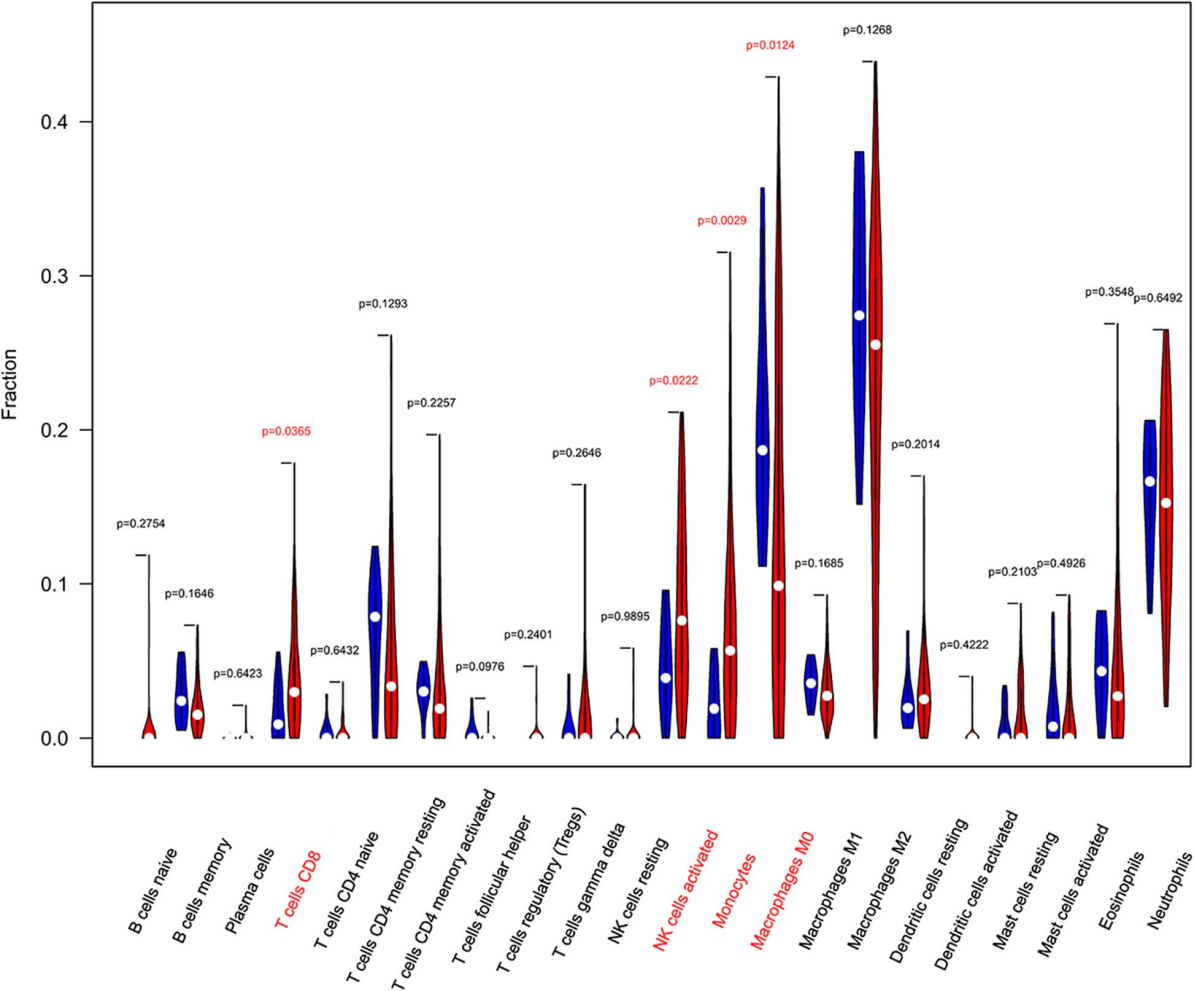
Violin diagram of the proportion of 22 kinds of immune cells in asthma and control groups. Blue and red colors represent control and asthma samples, respectively. Markers in red indicate significant differences between the two groups

**Fig. 10:**
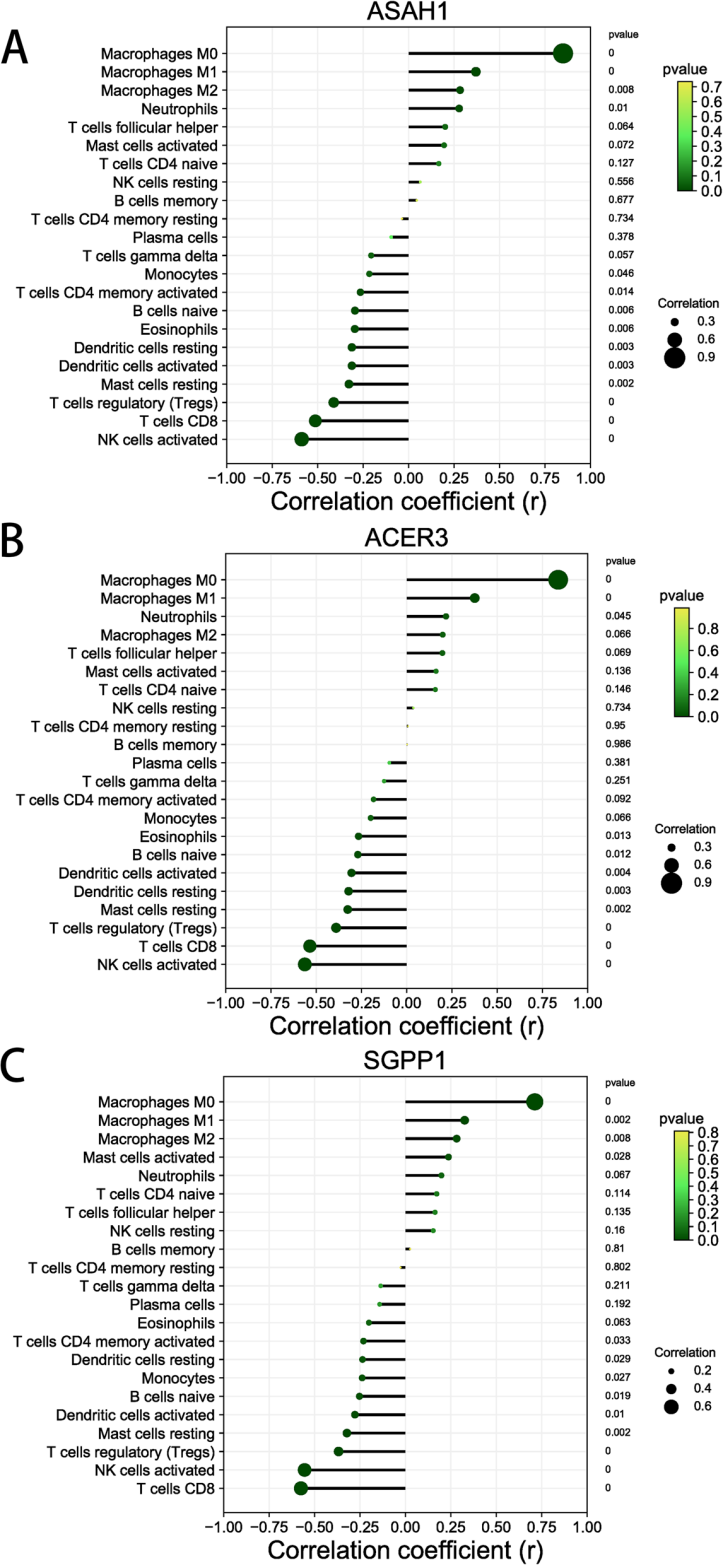
Correlation analysis between hub genes and 22 immune cells. **A**-**C** The lollipop diagram showing the relationship of hub genes (*ASAH1*, *ACER3* and *SGPP1*) and immune cells

**Fig. 11:**
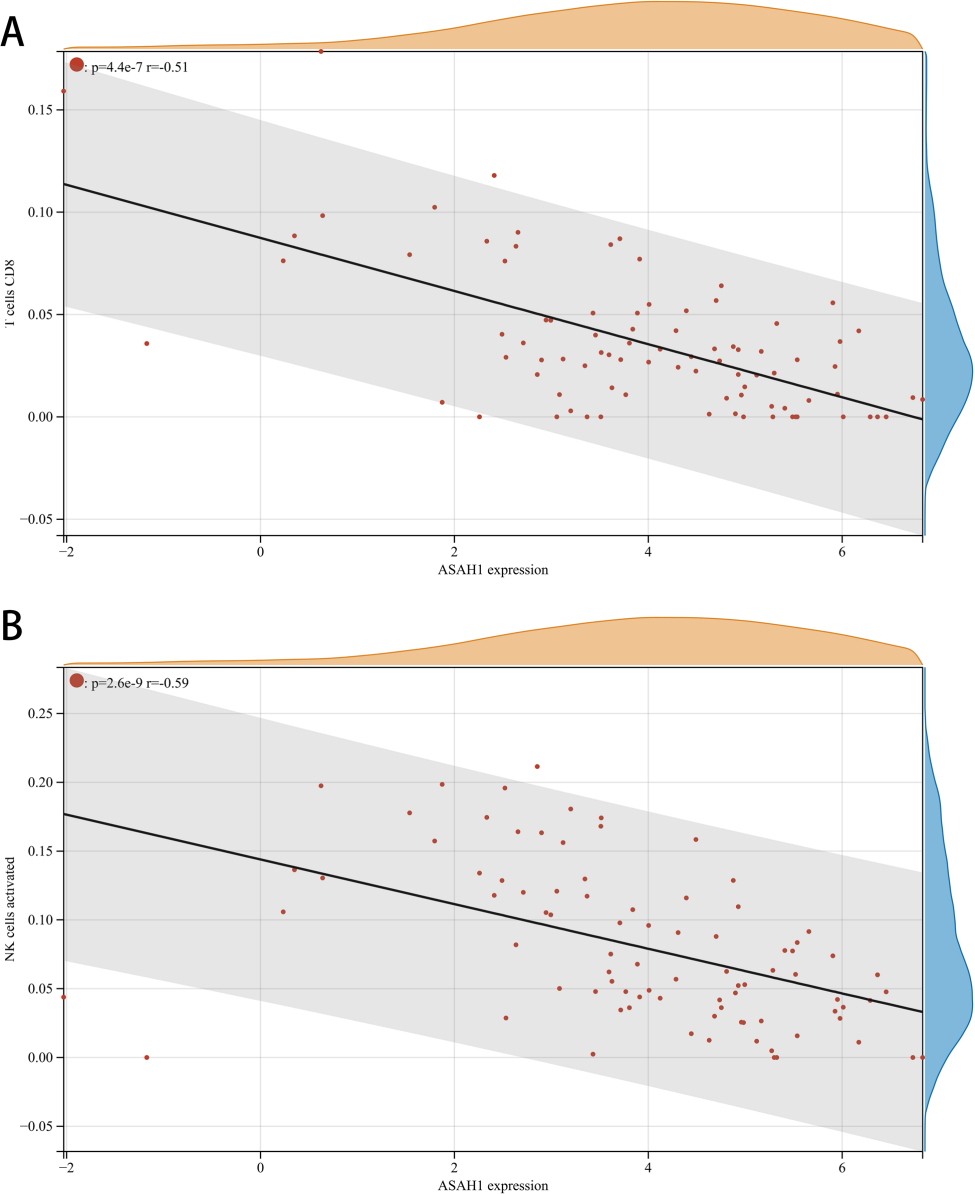
Correlation of *ASAH1* with differentially-expressed immune cells. **A**, **B** Association between *ASAH1* and CD8 + T cell and activated NK cells

**Fig. 12:**
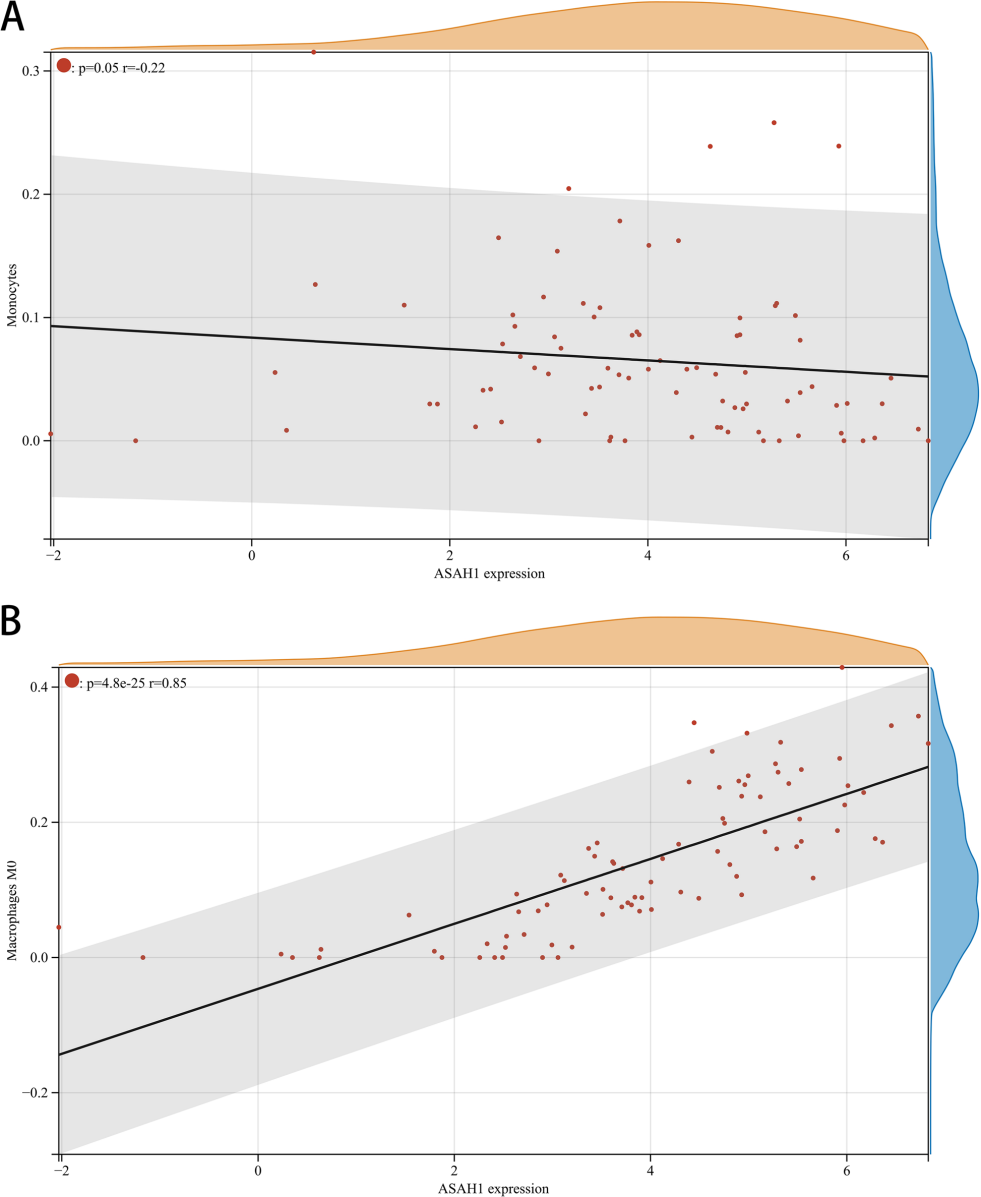
Correlation of *ASAH1* with differentially-expressed immune cells. **A**, **B** Association between *ASAH1* and monocytes and M0 macrophages

**Fig. 13:**
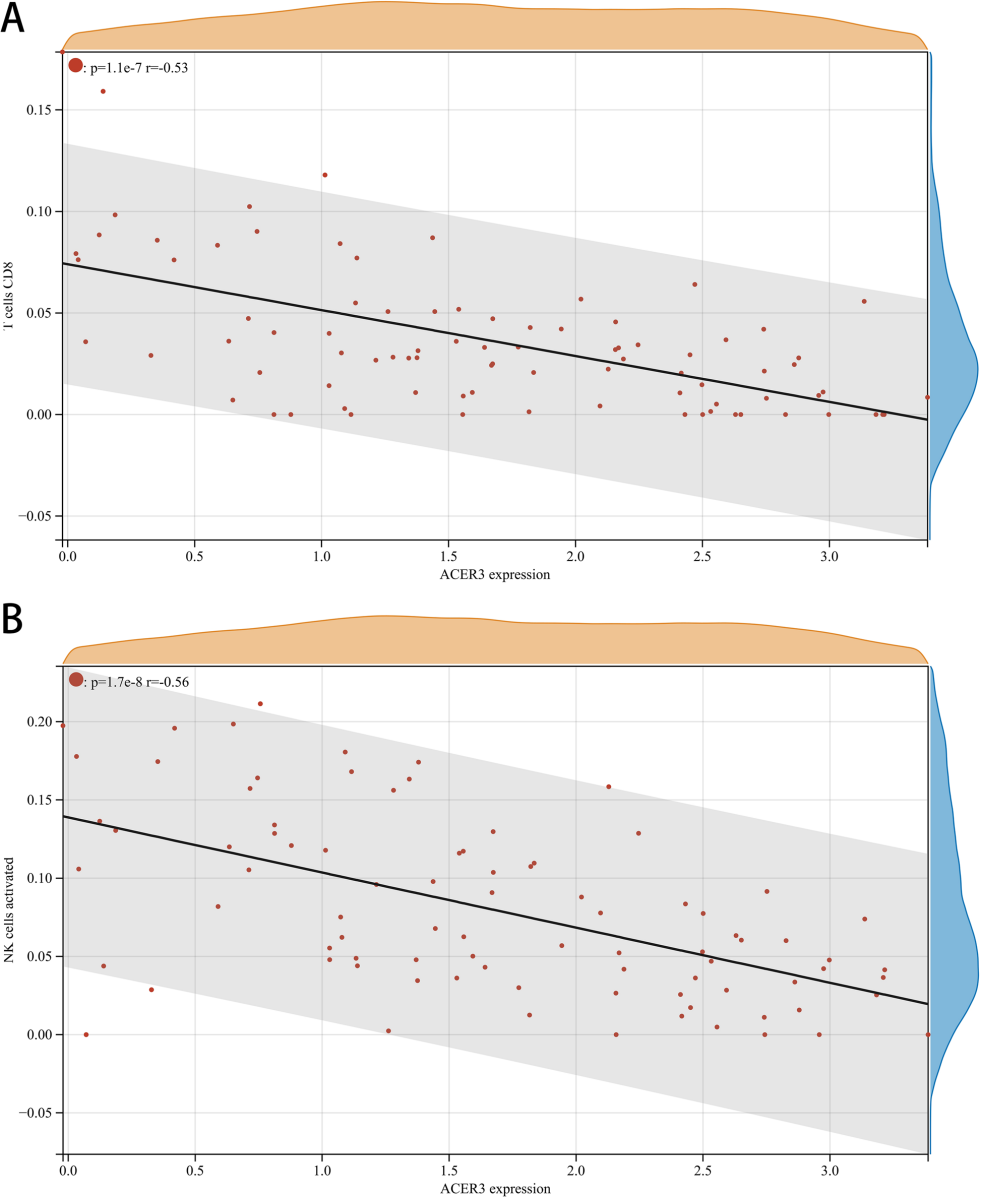
Correlation of *ACER3* with differentially-expressed immune cells. **A**, **B** Association between *ACER3* and CD8 + T cell and activated NK cells

**Fig. 14:**
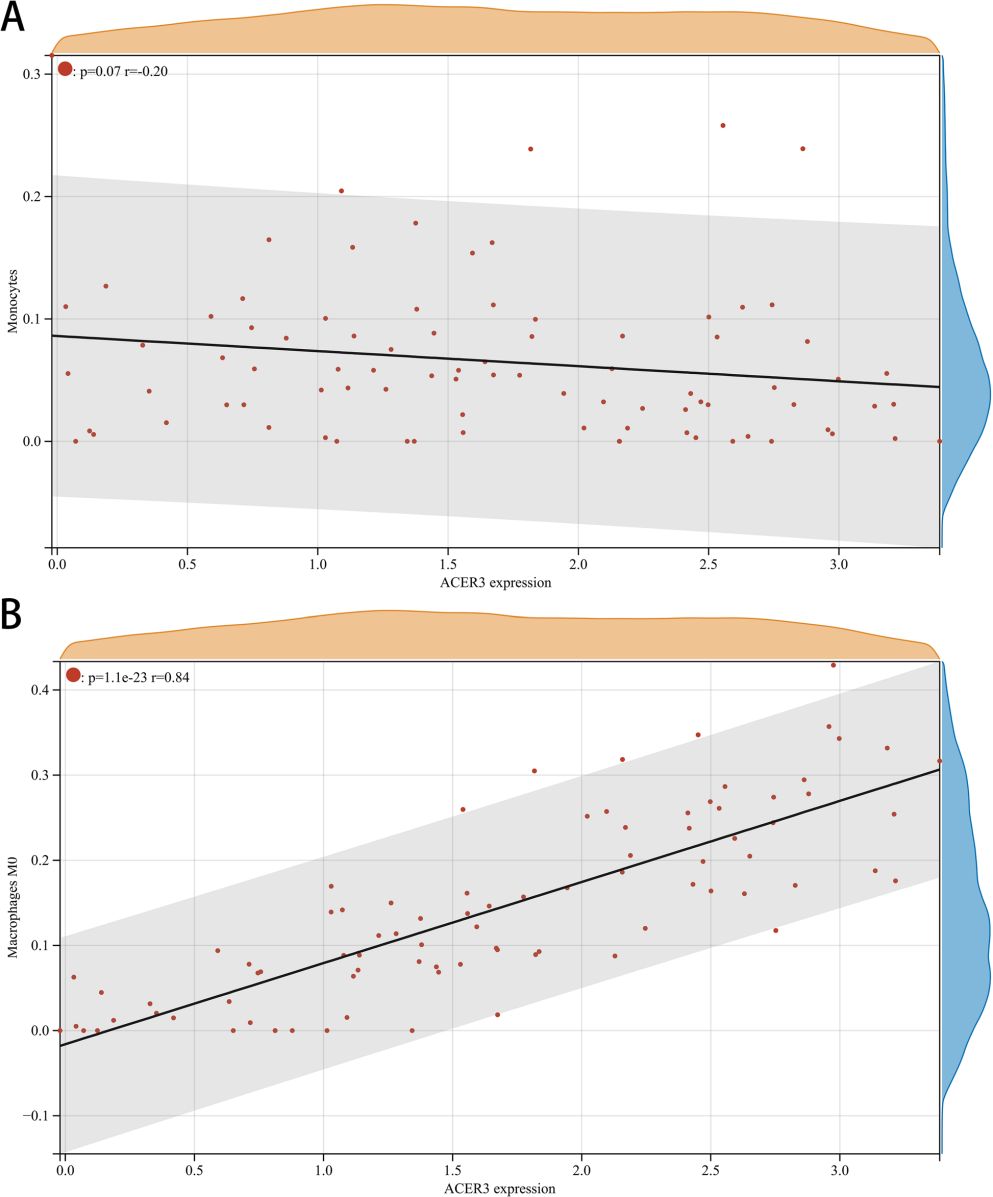
Correlation of *ACER3* with differentially-expressed immune cells. **A**, **B** Association between *ACER3* and monocytes and M0 macrophages

**Fig. 15:**
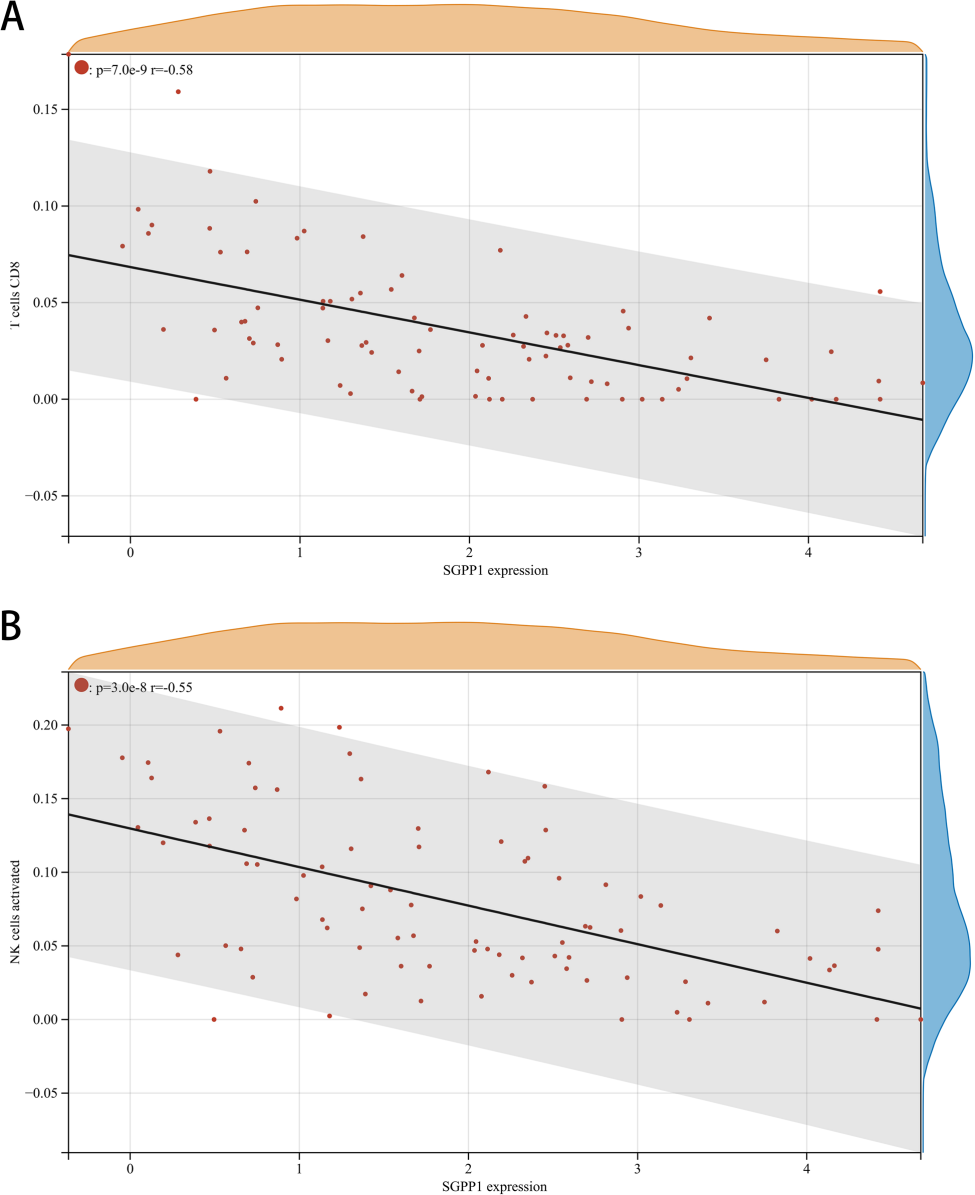
Correlation of *SGPP1* with differentially-expressed immune cells. **A**, **B** Association between *SGPP1* and CD8 + T cell and activated NK cells

**Fig. 16:**
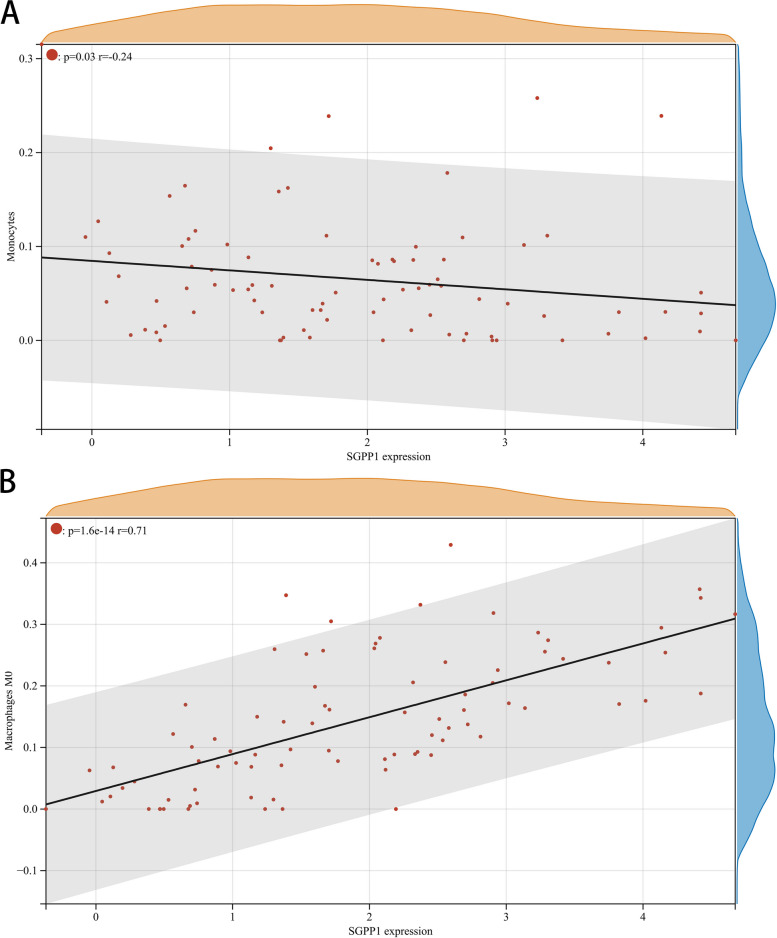
Correlation of *SGPP1* with differentially-expressed immune cells. **A**, **B** Association between *SGPP1* and monocytes and M0 macrophages

The proportion and expression of 22 immune cells in each sample were clearly visualized in the bar plot and heatmap (Fig. 8A, B). The violin diagram demonstrated that CD8^+^ T cells, activated NK cells, and monocytes were highly expressed in the asthma group compared with the control group, whereas M0 macrophages showed less expression (Fig. 9). The correlation analysis between diagnostic biomarkers and immune cells revealed that *ASAH1*, *ACER3*, and *SGPP1* were negatively correlated with CD8^+^ T cells and activated NK cells but positively correlated with M0 macrophages. Moreover, *SGPP1* showed a negative relationship with monocytes (Figs. 10A-C, 11A, B, 12A, B, 13A, B, 14A, B, 15A, B and 16A, B). Therefore, these results demonstrated that CD8^+^ T cells, activated NK cells, monocytes, and M0 macrophages may be the potential core immune cells involved in the pathogenesis of asthma, and the hub genes are correlated with the immune infiltration landscape, which might provide fresh insight for future research on asthma.

### Construction of the ceRNA network

**Fig. 17:**
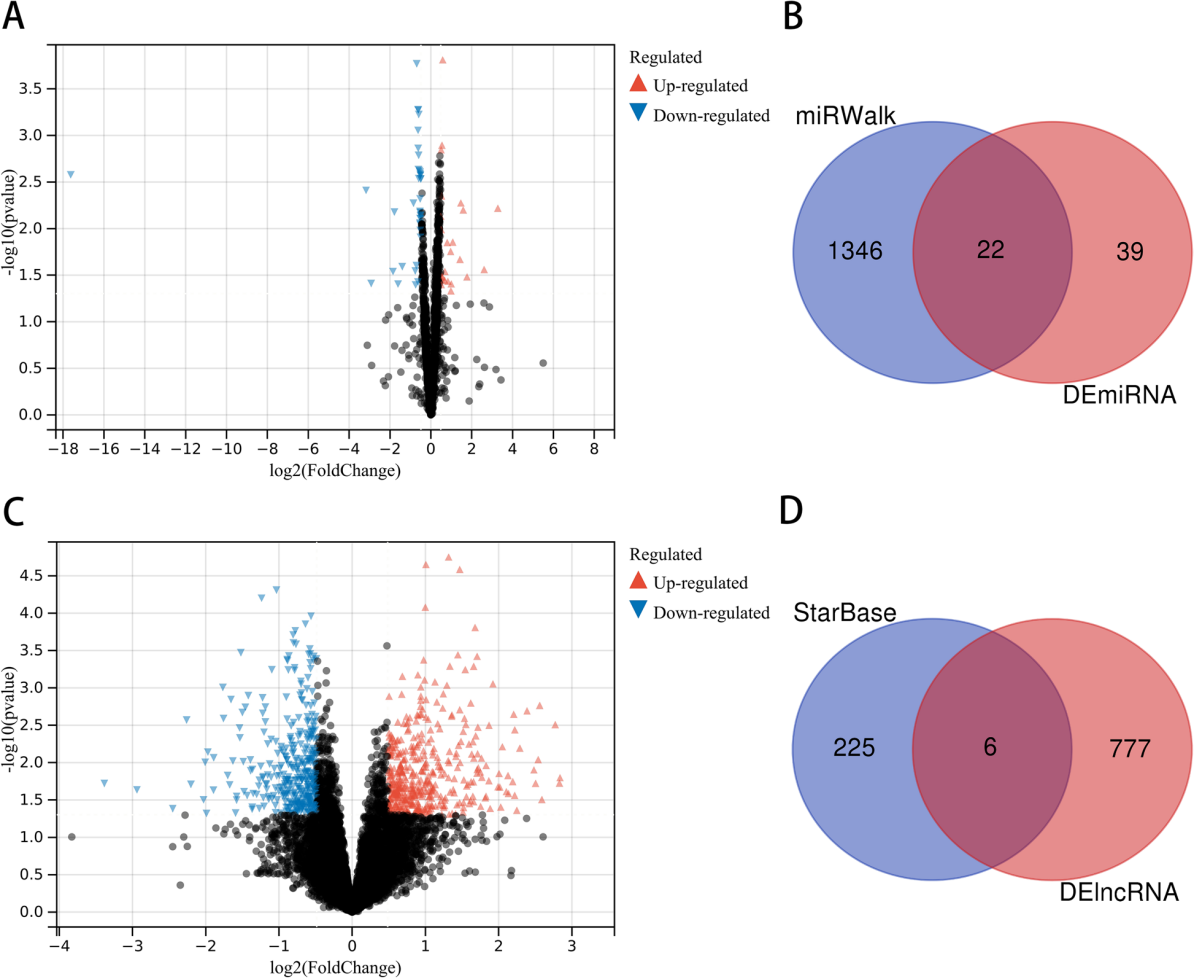
CeRNA network construction based on hub genes. **A** Volcano plot showing DEmiRNA in asthma and control groups. **B** Venn diagram showing common miRNA between miRWalk and DEmiRNA. **C** Volcano plot showing DElncRNA in asthma and control groups. **D** Venn diagram showing common miRNA between StarBase and DElncRNA. DEmiRNA: Differentially expressed miRNAs, DElncRNA: differentially expressed lncRNA

**Fig. 18:**
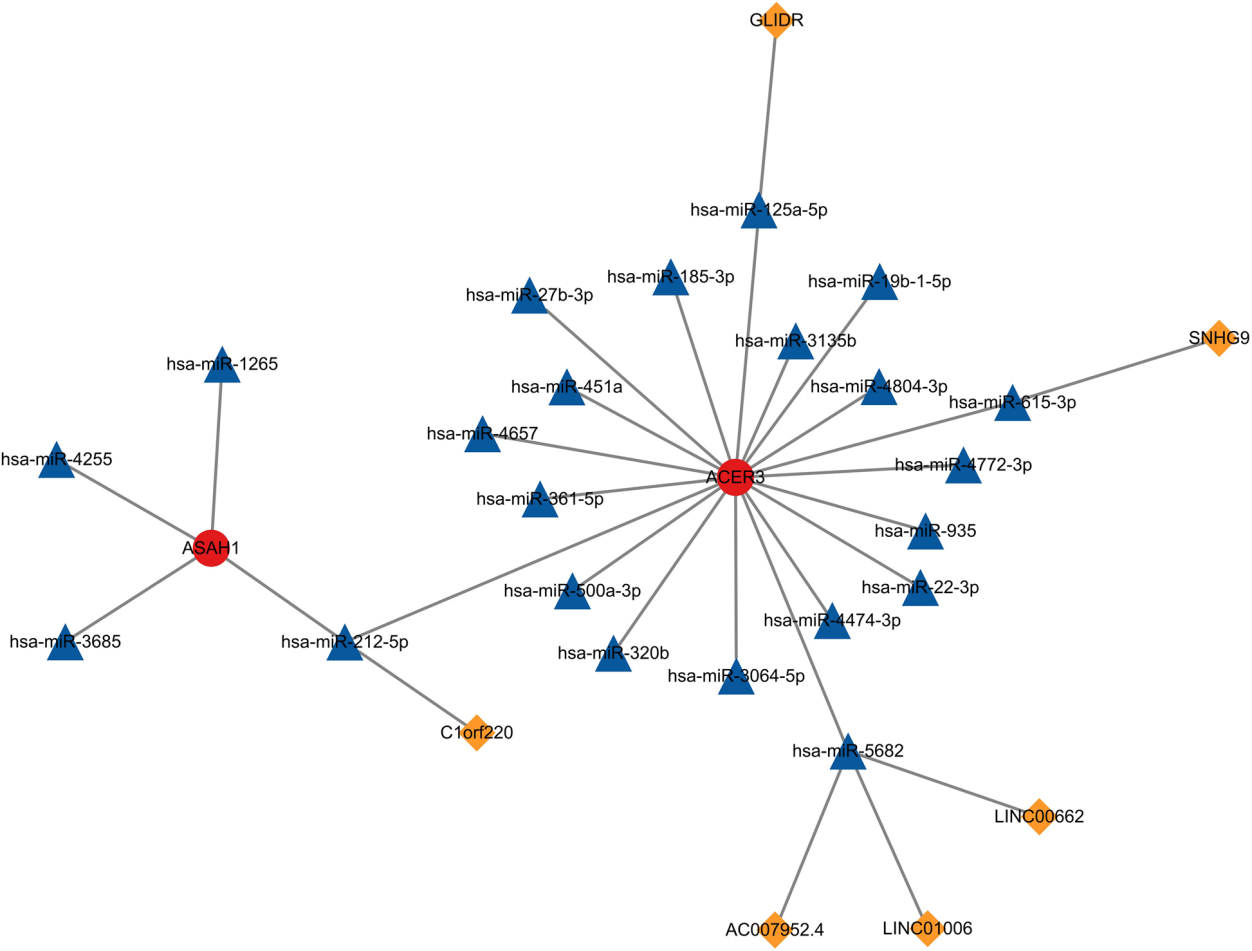
The ceRNA network

We selected 61 DEmiRNAs from the GSE120172 dataset, including 23 upregulated miRNAs and 38 downregulated miRNAs (Fig. 17A). miRWalk was utilized to predict the target miRNAs of three hub genes (*ASAH1*, *ACER3* and *SGPP1*). A total of 1368 miRNAs were selected, and the potential miRNAs were intersected with 61 DEmiRNAs to obtain 22 common miRNAs (Fig. 17B). Then, 783 DElncRNAs were selected from the GSE143192 dataset, including 426 upregulated lncRNAs and 357 downregulated lncRNAs (Fig. 17C). The starBase database was utilized to predict lncRNAs interacting with the common miRNAs. We obtained 231 lncRNAs, and 6 lncRNAs overlapped between 231 predicted lncRNAs and 783 DEmiRNAs (Fig. 17D). Finally, a network of 2 hub genes, 22 miRNAs, and 6 lncRNAs was established (Fig. 18). Within the network, downregulated *SNHG9* may function as a ceRNA to inhibit *ACER3* by activating *hsa-miR-615-3p*. In addition, the upregulation of *hsa-miR-4255*, *hsa-miR-1265*, and *hsa-miR-3685* may suppress the expression of *ASAH1*. Notably, *hsa-miR-212-5p* might regulate *ASAH1* and *ACER3* at the same time, and *hsa-miR-5682* could bind to three lncRNAs (LINC00662, LINC01006, and AC007952.4); thus, *hsa-miR-212-5p* and *hsa-miR-5682* may be particularly important in the ceRNA network.

### Validation of hub genes

**Fig. 19:**
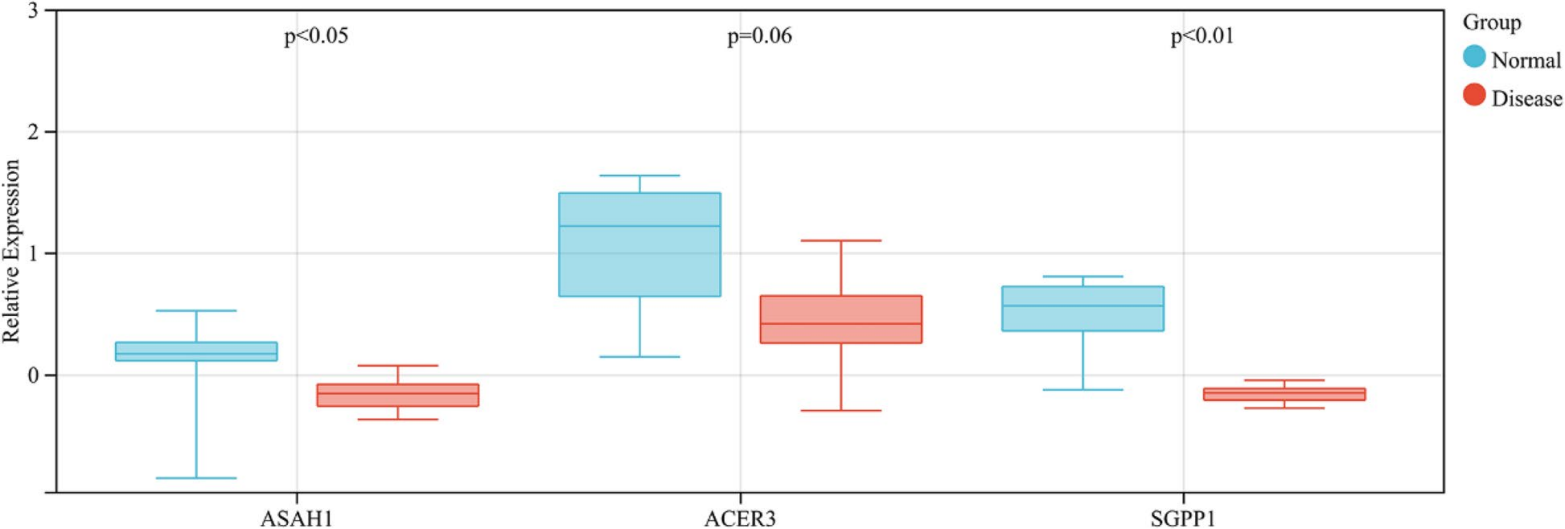
Validation of hub Genes in the GSE74075 dataset

The expression distributions of *ASAH1*, *ACER3* and *SGPP1* were validated in the GSE74075 dataset. The Wilcoxon test results demonstrated that the expression levels of *ASAH1* (*P* < 0.05) and *SGPP1* (*P* < 0.01) were also significantly downregulated in asthmatic patients compared with those in healthy controls, which were similar to those in GSE74986. Interestingly, *ACER3* (*P* = 0.06) was also downregulated in GSE74075, but the difference was not statistically significant (Fig. 19). Therefore, *ASAH1* and *SGPP1* may be the key genes involved in the occurrence of asthma.

**Fig. 20:**
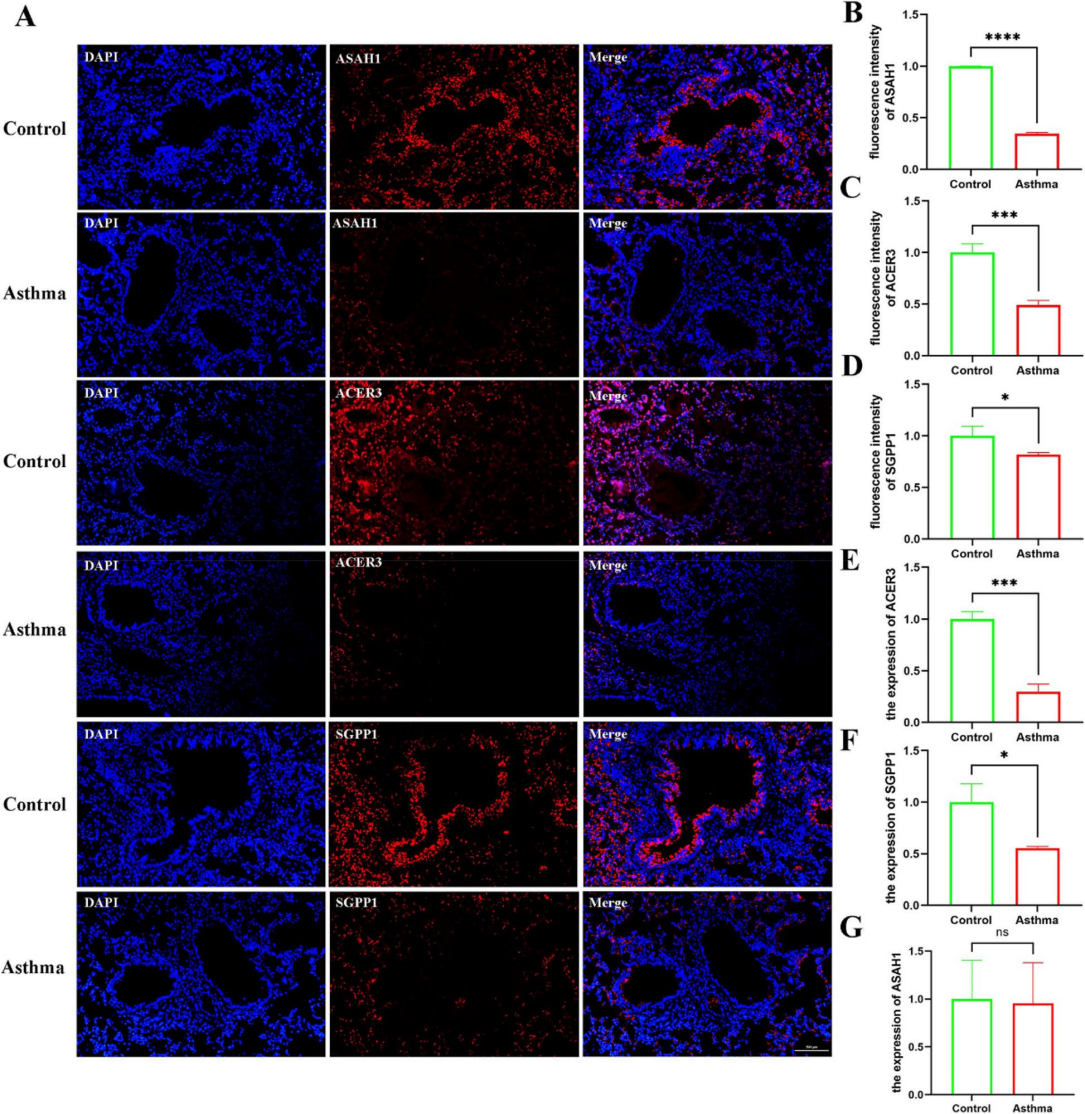
The result of validation by immunofluorescent staining and RT-qPCR in mouse models (**A**–**D**) immunofluorescent staining images of lung tissues and quantification of fluorescence intensity, Nucleus (blue), ASAH1/ACER3/SGPP1 (red), scale bar, 500 um, (*n* = 4 per group) (**E**–**G**) The expression of *ASAH1*, *ACER3*, and *SGPP1* verified by RT- qPCR in the OVA model (*n* = 4 per per)

At the protein level, immunofluorescence staining showed that *ASAH1*, *ACER3* and *SGPP1* had lower expression levels in the OVA group than in the control group (*P* < 0.05) (Fig. 20A-D). RT‒qPCR showed that *ASAH1*, *ACER3* and *SGPP1* mRNA were downregulated in OVA mice. There was a statistically significant difference in *ACER3* and *SGPP1* (*P* < 0.05), which is consistent with previous results (Fig. 20E-G).

### Validation of the ceRNA network

The predicted target lncRNAs, miRNAs and the hub genes in the ceRNA network were examined in asthma cellular models. RT-qPCR results showed that *SNHG9*, *AC007952.4* and *ACER3* were significantly decreased in the IL-13-induced asthma cellular model than in the control group, while *hsa-miR-125a-5p*, *hsa-miR-615-3p* showed high expression (*P* < 0.05). The differences in the other lncRNAs, miRNAs and *ASAH1* between the two groups were not statistically significant (Additional file 6: Fig. S1). Only the *SNHG9-hsa-miR-615-3p-ACER3* axis was consistent with the relationship in the ceRNA network. To further verify this network, BEAS-2B cells were transfected with the small interfering RNA (siRNA) in the subsequent experiments. After *SNHG9* knockdown, lower level of the *SNHG9* was seen in BEAS-2B cells, confirming the efficiency of siRNA transfection, with a corresponding decrease in *ACER3* expression, while *hsa-miR-615-3p* level increased in the cells (Additional file 6: Fig. S2). The above results confirmed the reliability of the *SNHG9-hsa-miR-615-3p-ACER3* network.

## Discussion

Lipid metabolism is a novel hallmark of asthma, and the involvement of LMRGs in the initiation, progression, and treatment of asthma has gained increasing attention [33–35]. To the best of our knowledge, this is the first study to comprehensively explore the roles of LMRGs in combination with the immune microenvironment in the pathogenesis of asthma by conducting WGCNA and immune infiltration analysis and constructing a ceRNA network. Our findings may facilitate the development of targeted therapy for asthma.

In this study, a total of 520 DEGs were selected, and 1431 genes in the turquoise module most related to asthma with a strong GS-MM correlation were identified through WGCNA. We then intersected these genes with 769 LMRGs to obtain 32 BA-LM DEGs. Enrichment analysis showed that the BA-LM DEGs were mainly involved in the PPAR signaling pathway, sphingosine metabolic process, and inositol phosphate metabolism, suggesting that metabolic pathways may mediate the role of LMRGs in the pathogenesis of asthma. Peroxisome proliferator-activated receptors (PPARs) are ligand-activated transcription factors belonging to the nuclear hormone receptor superfamily, which can regulate several metabolic pathways and may possess potent anti-inflammatory and immunomodulatory activity [36]. The knockout of the PPARα gene could increase the disease phenotype in an allergic asthma model [37]. In addition, inositol phosphate metabolism could increase muscle contraction of hyperresponsive tracheas and is associated with smooth muscle function [38, 39]. Metabonomics studies also found that it was an important mediator involved in bronchial asthma [40, 41]. Thus, activating PPARs and inhibiting inositol phosphate metabolism may be potential novel treatments for asthma [36, 42].

Three downregulated LMRGs (*ASAH1*, *ACER3* and *SGPP1*) were identified as hub genes for asthma, which were validated in GSE143192. Meanwhile, RT‒qPCR and immunofluorescence analysis were performed to verify the results at the mRNA and protein levels and further enhance the reliability of the study. *ASAH1* (N-acylsphingosine amidohydrolase 1), located on chromosome 8p22, encodes a member of the acid ceramidase family of proteins [43], which hydrolyses ceramide into sphingosine and free fatty acids [43, 44]. Ceramide is a sphingolipid with powerful proinflammatory and proapoptotic properties. Previous studies have demonstrated that elevation of ceramide levels contributes to the development of airway inflammation and dysfunction and hyperresponsiveness in asthma [45, 46]. Moreover, ceramide metabolism could be a potential anti-rhinoviral target involved in acute worsening of asthma [47], and ceramide/sphingosine-1-phosphate imbalance was an underlying metabolic signature among asthmatic patients [46], indicating that *ASAH1* may be involved in the pathogenesis of asthma. GSEA revealed that the role of *ASAH1* in asthma may be closely related to protein secretion, adipogenesis, mTORC1 signaling, fatty acid metabolism, and E2F targets. Therefore, *ASAH1* might be a potential novel biomarker to diagnose and treat asthma.

*ACER3* (alkaline ceramidase 3), located on chromosome 11q13.5, is mostly enriched in the superpathway of sphingolipid metabolism [48]. Similar to *ASAH1*, *ACER3* is one of three alkaline ceramidases (ACERs) that catalyze the conversion of ceramide to sphingosine. Furthermore, sphingolipids, as bioactive molecules, are consistently implicated in lung inflammation and airway hyperreactivity, and genetically altered sphingolipid metabolism could affect airway resistance and may predispose patients to the development of asthma [49]. These findings may indicate that *ACER3* might play a pivotal role in asthma pathogenesis. While not statistically significant, *ACER3* was also downregulated in asthmatic patients within the GSE74075 dataset. The specific role of *ACER3* in asthma has not been reported, and future studies are required to further explore the connection between *ACER3* and asthma.

We observed significantly reduced *SGPP1* expression levels in asthma patients. *SGPP1* (sphingosine-1-phosphate phosphatase 1), located on chromosome 14q23.2, is a member of the type 2 lipid phosphate phosphatase family. Although there is no relevant study on *SGPP1* in asthma, it has been reported that *SGPP1* could regulate the intracellular level of sphingosine-1-phosphate (S1P) [50], which is a biomarker, pathogenic contributor, and therapeutic target for asthma [51, 52]. In addition, among its related superpathway is sphingolipid metabolism [53]. These findings may support the possible role of *SGPP1* in the pathogenesis of asthma. Furthermore, GSEA indicated that *SGPP1* might be mainly involved in fatty acid metabolism, protein secretion, E2F targets, mTORC1 signaling, and MYC target v1. Nevertheless, the role of *SGPP1* in asthma needs to be further confirmed.

Further analysis showed that three downregulated LMRGs (*ASAH1*, *ACER3* and *SGPP1*) were negatively correlated with CD8^+^ T cells, activated NK cells and monocytes and positively correlated with M0 macrophages, suggesting that downregulated hub genes may be associated with increased immune system activation in asthma patients. Previous studies showed that knockout of the *ASAH1* gene elevated ceramide levels resulting in enhanced cytotoxic activity of CD8 + T cells [54], and *ASAH1* and *ACER3* were closely connected with sphingolipid signaling [55, 56], which is involved in regulating the functions of immune cells such as CD4 + T cells, CD8 + T cells, NK cells, and macrophages [57]. Thus, we hypothesized that individuals with downregulated LMRGs (*ASAH1*, *ACER3* and *SGPP1*) may be liable to mediate the immune response, and more easily develop asthma. However, further research is needed to clarify the complex interactions between LMRGs and immune cells.

The ceRNA network suggested that *SNHG9*-*hsa-miR-615-3p*-*ACER3* may be implicated in asthma. Researchers found that overexpression of *SNHG9* alleviated inflammation and apoptosis of endothelial cells by suppressing TRADD expression [58] and was correlated with increased immune infiltrates [59]. Wang et al [60]. discovered that the gut microbiota reprogramed intestinal lipid metabolism through long noncoding RNA *SNHG9*. Hence, we speculated that *SNHG9* might paly an unanticipated role in lipid metabolism in asthma. However, the role of *SNHG9* and *hsa-miR-615-3p* in asthma has not been reported in previous studies. Overall, *SNHG9*, *hsa-miR-615-3p* and *ACER3* might be viewed as effective therapeutic targets for asthma.

Finally, although this study comprehensively found the essential roles of LMRGs and the immune microenvironment in asthma pathogenesis, some limitations should also be noted. First, the clinical information contained in the existing data is limited, so we could not control the effect of different factors on the results. Second, the GEO database did not have enough samples, which may cause statistical error. We validated the low expression of *ASAH1*, *ACER3* and *SGPP1* in the GSE74075 dataset. In addition, RT‒qPCR and immunofluorescence analysis verified the results at the mRNA and protein levels further enhancing the reliability of the study.

## Conclusions

In summary, we found a number of LMRGs and related signaling pathways in asthma. Among these genes, *ASAH1*, *ACER3*, and *SGPP1* were identified as potential diagnostic biomarkers for asthma. CD8^+^ T cells, activated NK cells, monocytes, and M0 macrophages might be the critical immune cells implicated in asthma, and we conjectured that people with downregulated hub genes may be liable to mediate the immune response, and more easily develop asthma. In addition, *SNHG9*-*hsa-miR-615-3p*-*ACER3* might be involved in asthma pathogenesis, and *hsa-miR-212-5p* and *hsa-miR-5682* could be viewed as effective therapeutic targets for asthma. Our findings might provide new perspectives and insights for future research on asthma.

### Supplementary Information

**Additional file 1: Table S1. **Detailed information of the studied gene expression profiles. Table S2. The characteristics of participants in the GSE74075.

**Additional file 2: Table S3. **Real-time quantitative PCR primer sequences.

**Additional file 3: Table S4. **Volcano plot upregulated gene dataset.

**Additional file 4: Table S5. **Volcano plot downregulated gene dataset.

**Additional file 5: Table S6. **Genes in the turquoise module.

**Additional file 6: Fig. S1. **The expression level of predicted target lncRNAs, miRNAs and the hub genes in IL-13-induced BEAS-2B cells measured by qRT-PCR. **Fig. S2.** The expression level of SNHG9, hsa-miR-615-3p and ACER3 in each group after siRNA transfection.

## Data Availability

The datasets analysed during the current study are available in GEO datasets GSE74986 (https://www.ncbi.nlm.nih.gov/geo/query/acc.cgi?acc = GSE74986), GSE74075 (https://www.ncbi.nlm.nih.gov/geo/query/acc.cgi?acc = GSE74075), GSE120172 (https://www.ncbi.nlm.nih.gov/geo/query/acc.cgi?acc = GSE120172), and GSE143192 (https://www.ncbi.nlm.nih.gov/geo/query/acc.cgi?acc = GSE143192).
