# Supplementary material for: Lipid metabolism-related genes are involved in the occurrence of asthma and regulate the immune microenvironment
Source: BMC Genomics. 2024 Feb 1;25:129. doi: 10.1186/s12864-023-09795-3 (PMC10832186; doi:10.1186/s12864-023-09795-3)
Supplement: Supplementary file 1 — Additional file 1: Table S1. Detailed information of the studied gene expression profiles. Table S2. The characteristics of participants in the GSE74075. [file 12864_2023_9795_MOESM1_ESM.docx]

| Additional file 1. Table S1. Detailed information of the studied gene expression profiles. | | | | | | | | |
| --- | --- | --- | --- | --- | --- | --- | --- | --- |
| Dataset | Platform | Control (n) | Asthma (n) | Samples | Application | Author | Country | Submission |
| GSE74986 | GPL6480 Agilent-014850 Whole Human Genome Microarray 4x44K G4112F | 12 | 74 | Bronchial alveolar lavage | Identification for DEmRNAs | Sun Y^1^ | USA | 2015 |
| GSE120172 | GPL25586 Exiqon miRCURY LNA microRNA array | 12 | 12 | Peripheral blood | Identification for DEmiRNAs | Tian J^2^ | China | 2021 |
| GSE143192 | GPL22120 Agilent-078298 human ceRNA array V1.0 4X180K | 4 | 4 | Peripheral blood | Identification for DElncRNAs | Xia L^3^ | China | 2021 |
| GSE74075 | GPL6883 Illumina HumanRef-8 v3.0 expression beadchip | 6 | 10 | Sputum | Validation for key biomarkers | Li Q^4^ | Australia | 2016 |

*Abbreviations*: *n* Number, *DEmiRNAs* Differentially expressed miRNAs, *DEmRNAs* Dfferentally expressed mRNAs, *DElncRNAs* Differentially expressed lncRNA.

| Additional file 1. Table S2. The characteristics of participants in the GSE74075. | | | |
| --- | --- | --- | --- |
| Characteristics | | Healthy | Asthma |
| *N* |  | 6 | 10 |
| Age (y), mean±SD |  | 37.67±8.33 | 50.00±6.57 |
| Gender, n (%) | Male | 4(66.67%) | 6(60.00%) |
|  | Female | 2(33.33%) | 4(40.00%) |
| BMI (kg/m^2^), mean±SD |  | 32.09±1.24 | 28.51±0.15 |
| Smoking, n (%) | Nonsmoker | 2(33.33%) | 32(76.19%) |
|  | Former smoker | 4(66.67%) | 10(23.81%) |

*Abbreviations*: *n* Number, *BMI* Body Mass Index.

**References**

1. Sun, Y.; Peng, I.; Webster, J. D.; Suto, E.; Lesch, J.; Wu, X.; Senger, K.; Francis, G.; Barrett, K.; Collier, J. L.; et al. Inhibition of the kinase ITK in a mouse model of asthma reduces cell death and fails to inhibit the inflammatory response. *Science signaling* **2015,** *8* (405), ra122.

2. Li, Q.; Baines, K. J.; Gibson, P. G.; Wood, L. G. Changes in Expression of Genes Regulating Airway Inflammation Following a High-Fat Mixed Meal in Asthmatics. *Nutrients* **2016,** *8* (1).

3. Tian, J.; Ning, J.; Xu, Y. [Bioinformatics analysis of differentially expressed microRNAs in children with bronchial asthma]. *Chinese journal of cellular and molecular immunology* **2021,** *37* (10), 923-931.

4. Xia, L.; Wang, X.; Liu, L.; Fu, J.; Xiao, W.; Liang, Q.; Han, X.; Huang, S.; Sun, L.; Gao, Y.; et al. lnc-BAZ2B promotes M2 macrophage activation and inflammation in children with asthma through stabilizing BAZ2B pre-mRNA. *The Journal of allergy and clinical immunology* **2021,** *147* (3), 921-932.e9.
