# Supplementary material for: Lipid metabolism-related genes are involved in the occurrence of asthma and regulate the immune microenvironment
Source: BMC Genomics. 2024 Feb 1;25:129. doi: 10.1186/s12864-023-09795-3 (PMC10832186; doi:10.1186/s12864-023-09795-3)
Supplement: Supplementary file 2 — Additional file 2: Table S3. Real-time quantitative PCR primer sequences. [file 12864_2023_9795_MOESM2_ESM.docx]

Additional file 2. Table S3. Real-time quantitative PCR primer sequences.

| Symbol | Primer Sequence |
| --- | --- |
| *GLIDR* | forward: 5'-GGGTGACTGCTCTCCATGTT-3' |
|  | reverse: 5'-CTTCTCCAGGCAACAGCCAA-3' |
| *SNHG9* | forward: 5'-GAATCCACCCGAAGAGTGGC-3' |
|  | reverse: 5'-ACCAGTGTCCTAAGTGAAGAGG-3' |
| *LINC00662* | forward: 5'-CGCCCTGTGACTAACGG-3' |
|  | reverse: 5'-AAAGCAGGATTGTAGGGCG-3' |
| *LINC01006* | forward: 5'- CACCACCACAAAGCCTCAAG-3' |
|  | reverse: 5'-CCCATACTAGGAGAAACATGGG-3' |
| *AC007952.4* | forward: 5'-CTGGAAGGTGGGACTTGCTC-3' |
|  | reverse: 5'-GTTTACAGCGTGGCTGATGC-3' |
| *C1orf220* | forward: 5'-AGATGCCTCCCAGCATAAGG-3' |
|  | reverse: 5'-GGGCCAAGACATACCCAGG-3' |
| *hsa-miR-212-5p* | forward: 5'-TGGACGATACCTTGGCTCTAGAC-3' |
|  | reverse: 5'-TATCCTTCTTCACGACTCCTTCAC-3' |
| *hsa-miR-5682* | forward: 5'-AATGAGTCGTAGCACCTTGCAG-3' |
|  | reverse: 5'-TATCCTTGTTGACGACTGGTTGAC-3' |
| *hsa-miR-615-3p* | forward: 5'-ACGGTCCGAGCCTGGGT-3' |
|  | reverse: 5'-TATGGTTGTTCACGACTCCTTCAC-3' |
| *hsa-miR-125a-5p* | forward: 5'-ACTACATCCCTGAGACCCTTTAAC-3' |
|  | reverse: 5'-TATGGTTTGACGACTGTGTGAT-3' |
| *GAPDH* | forward: 5'-GGCCCCTCTGGAAAGCTGTGG-3' |
|  | reverse: 5'-CCCGGCATCGAA GGT-GGAAGA-3' |
| *U6* | forward: 5'-CAGCACATATACTAAAATTGGAACG-3' |
|  | reverse: 5'-ACGAATTCGTGTCATCC-3' |
