# Supplementary material for: Lipid metabolism-related genes are involved in the occurrence of asthma and regulate the immune microenvironment
Source: BMC Genomics. 2024 Feb 1;25:129. doi: 10.1186/s12864-023-09795-3 (PMC10832186; doi:10.1186/s12864-023-09795-3)
Supplement: Supplementary file 6 — Additional file 6: Fig. S1. The expression level of predicted target lncRNAs, miRNAs and the hub genes in IL-13-induced BEAS-2B cells measured by qRT-PCR. Fig. S2. The expression level of SNHG9, hsa-miR-615-3p and ACER3 in each group after siRNA transfection. [file 12864_2023_9795_MOESM6_ESM.docx]

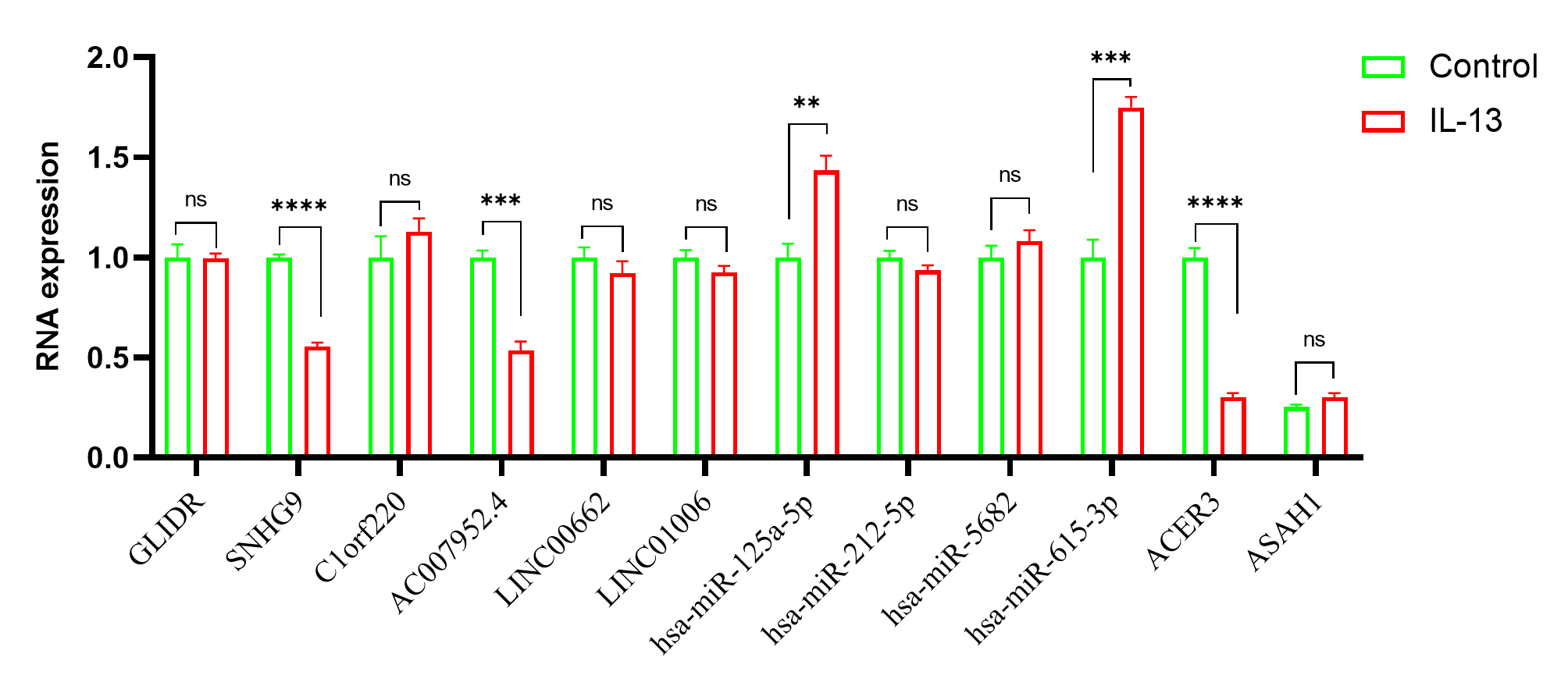


Additional file 6 Fig. S1. The expression level of predicted target lncRNAs, miRNAs and the hub genes in IL-13-induced BEAS-2B cells measured by qRT-PCR.


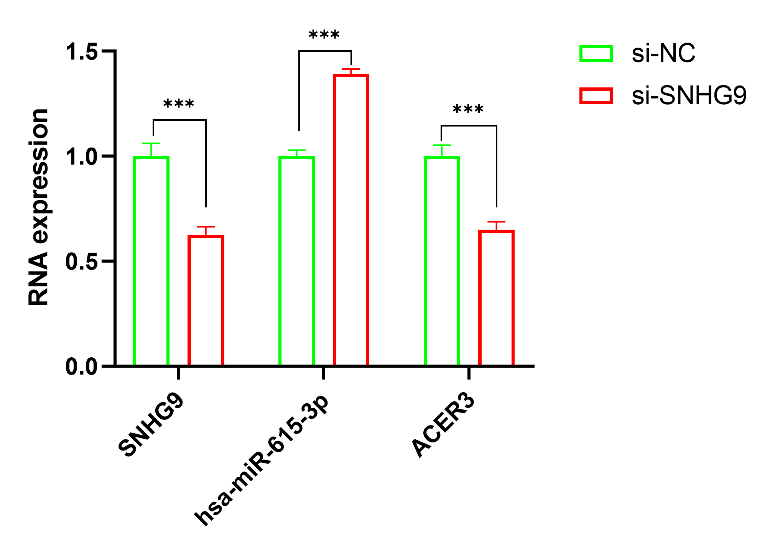


Additional file 6 Fig. S2. The expression level of *SNHG9*, *hsa-miR-615-3p and ACER3* in each group after siRNA transfection.
